# Linked read sequencing resolves complex genomic rearrangements in gastric cancer metastases

**DOI:** 10.1186/s13073-017-0447-8

**Published:** 2017-06-19

**Authors:** Stephanie U. Greer, Lincoln D. Nadauld, Billy T. Lau, Jiamin Chen, Christina Wood-Bouwens, James M. Ford, Calvin J. Kuo, Hanlee P. Ji

**Affiliations:** 1 Division of Oncology, Department of Medicine, Stanford University School of Medicine, CCSR 1115, 269 Campus Drive, Stanford, CA 94305-5151 USA; 2 Medical Oncology, Intermountain Healthcare, St. George, UT 84770 USA; 3 Stanford Genome Technology Center, Stanford University, Palo Alto, CA 94304 USA; 4 Division of Hematology, Department of Medicine, Stanford University School of Medicine, Stanford, CA 94305 USA

**Keywords:** Whole genome analysis, Cancer rearrangements, High molecular weight DNA, Cancer drivers, Barcode linked sequence reads

## Abstract

**Background:**

Genome rearrangements are critical oncogenic driver events in many malignancies. However, the identification and resolution of the structure of cancer genomic rearrangements remain challenging even with whole genome sequencing.

**Methods:**

To identify oncogenic genomic rearrangements and resolve their structure, we analyzed linked read sequencing. This approach relies on a microfluidic droplet technology to produce libraries derived from single, high molecular weight DNA molecules, 50 kb in size or greater. After sequencing, the barcoded sequence reads provide long range genomic information, identify individual high molecular weight DNA molecules, determine the haplotype context of genetic variants that occur across contiguous megabase-length segments of the genome and delineate the structure of complex rearrangements. We applied linked read sequencing of whole genomes to the analysis of a set of synchronous metastatic diffuse gastric cancers that occurred in the same individual.

**Results:**

When comparing metastatic sites, our analysis implicated a complex somatic rearrangement that was present in the metastatic tumor. The oncogenic event associated with the identified complex rearrangement resulted in an amplification of the known cancer driver gene *FGFR2*. With further investigation using these linked read data, the *FGFR2* copy number alteration was determined to be a deletion-inversion motif that underwent tandem duplication, with unique breakpoints in each metastasis. Using a three-dimensional organoid tissue model, we functionally validated the metastatic potential of an *FGFR2* amplification in gastric cancer.

**Conclusions:**

Our study demonstrates that linked read sequencing is useful in characterizing oncogenic rearrangements in cancer metastasis.

**Electronic supplementary material:**

The online version of this article (doi:10.1186/s13073-017-0447-8) contains supplementary material, which is available to authorized users.

## Background

Genomic rearrangements are alterations of large genomic segments, sometimes spanning megabases. Rearrangements are composed of structural variants (SVs), of which there are several classes, including large insertions, large deletions, inversions, duplications, and translocations. Germline SVs are a significant source of variation among normal genomes [1] while somatic SVs are widely observed among many different cancers [2, 3]. Somatic rearrangements of the cancer genome are important drivers of oncogenesis. For example, some translocations lead to oncogenic gain-of-function that can act as critical cancer drivers and potential therapeutic targets. One example is seen in chronic myelogenous leukemia, a hematologic malignancy, which is characterized by a translocation of chromosomes 9 and 22 that leads to the *BCR-ABL* chimeric gene product, an essential oncogenic driver [4, 5]. Similarly, cancers derived from solid tissues also have translocations that have functional significance in contributing to neoplastic development [6–9].

In tumors, genomic instability leads to somatic rearrangements. Detecting and characterizing these somatic rearrangements is particularly difficult due to the sheer structural complexity of cancer genomes [10]. Genomic instability can lead to complex combinations of multiple SVs that aggregate around specific loci [11, 12]. Determining the structure of cancer rearrangements is further complicated by the diploid nature of the human genome, since it is frequently unclear whether proximal SV events occur on the same parental chromosome (i.e., are in “*cis*”) or different chromosomes (i.e., are in “*trans*”). Adding to the difficulty of identifying somatic SVs, tumor cells rarely occur as pure cell populations in solid tumors, but are frequently intermingled with normal stroma. Thus, cancer genomes are practically “diluted” among normal diploid genomes and a somatic SV/rearrangement event may be represented in allelic fractions that are less than 50% of what one would see in a normal diploid genome [13, 14]. In this context, detection of somatic SVs from genomic DNA becomes more difficult. The use of RNAseq or other RNA-based molecular assays improves the sensitivity of detecting rearranged gene products [15, 16], but generally does not reveal the underlying structure of genomic DNA rearrangements.

Whole genome sequencing (WGS) with short sequence reads, typically less than several hundred bases, is the current method of choice for SV detection in cancer [17–19]. We will refer to whole genome analysis with short reads as conventional WGS. This approach has proven to be highly informative for characterizing cancer genomes in terms of genetic aberrations such as point mutations and presence of copy number alterations. However, using conventional WGS for SV discovery remains a significant challenge. This issue is a direct result of the molecular preparation necessary to generate short read data; high molecular weight (HMW) genomic DNA is fragmented into low molecular weight species, typically under 0.5 kb, and these short fragments are used to generate libraries for sequencing. As a result, one loses the genomic contiguity found in HMW DNA molecules. Without this genomic contiguity, it becomes significantly more difficult to determine structural changes that span larger, megabase-scale segments.

In conventional WGS, SV detection relies on a combination of the following methods: i) read count, ii) read-pair, iii) split-read, or iv) de novo assembly [18]. Performance among SV callers using short read sequence data varies significantly and independent verification is oftentimes required with a different type of molecular assay such as PCR amplicons that cross a novel breakpoint. These SV detection methods rely heavily on accurate read alignment—in highly repetitive regions of the genome, misalignment leads to a high rate of false positive SV calls. Moreover, with short read sequences derived from small fragments of DNA, it is extremely difficult to determine rearrangements that span megabase-scale segments and to reconstruct complex SV structures. Long read sequencing technologies, such as the Pacific Bioscience’s and Oxford Nanopore’s sequencers, generate reads on the scale of thousands of kilobases, and thus have seen application for SV detection and complex SV resolution [1]. However, the cost of these technologies is prohibitive for certain studies and the base qualities achieved are much lower than Illumina sequencing, which is an issue for complex samples where there are fractional allelic differences (Additional file 1: Table S1). For example, the high cost of long read sequencing technologies generally precludes their use for WGS and thus a targeted approach may be required, which relies on having prior knowledge of candidate complex SVs. In general, SV phasing and complex SV resolution is an ongoing area of research. Here, we use high quality sequence data derived from HMW DNA molecules with intact genomic contiguity to address issues associated with previous approaches.

We applied a recently developed library preparation technique that provides sequence from individual HMW DNA molecules to conduct a proof-of-concept study to identify somatic rearrangements from metastatic gastric tumors [20]. This technology relies on preparative microfluidics for generating droplet partitions. This process avoids any fragmentation and thus is ideal for sequencing HMW DNA molecules, on the order of 50 kb or higher. With an input of one nanogram of DNA that represents approximately 300 genome equivalents, the microfluidics distribute small amounts of input DNA across more than one million droplet partitions [20]. Each droplet contains anywhere from three to five DNA molecules along with a single gel bead reagent containing a unique oligonucleotide barcode that identifies each droplet (Additional file 2: Figure S1). In addition to the DNA and gel bead, each droplet contains the enzymatic reagents that enable random priming and polymerase amplification to occur. This process incorporates the droplet-specific barcode into the synthesized DNA. Barcode-tagged DNA molecules are released from the droplets and then undergo a final preparative step that results in complete libraries. Subsequently, the libraries are sequenced with an Illumina system.

Each paired-end read has a barcode identifier specific for a given droplet. One uses the barcode and mapping of the linked sequence reads to identify the originating HMW DNA molecule. Thus, the barcodes and linked reads directly reflect the identity and number of specific individual DNA molecules. The occurrence of DNA molecules with overlapping sequence is extremely small given that only three to five molecules are within each partition and the amount of input DNA is low. We used this molecular identification to characterize the HMW DNA molecules that span rearrangement breakpoints. In addition, the barcode linked reads enable one to phase *cis*-related genetic variants and identify larger haplotypes encompassing thousands of variants in megabase-size genomic segments. We used this haplotype information to make inferences about the relationship of SV breakpoints and infer the overall genomic structure of complex rearrangements occurring in cancer tissue samples. Previously, we demonstrated that linked read sequence data can be used to infer complex structural variants primarily based on visualizations [20]. In this study, we improve on the approach by quantifying barcode overlap between SV breakpoints, distinct SV events, and phased SNVs in order to assign SV phase. Further, identification of barcodes specific to SV events enables identification and fine-scale mapping of SV-specific reads to infer HMW structure and, thus, the structure of the original tumor DNA.

Gastric carcinoma is the fifth most common malignancy and the third leading cause of cancer deaths in the world [21]. Traditionally, gastric carcinoma has been classified into two subtypes—intestinal and diffuse—based on distinct histopathologic features. A recent genome survey of gastric carcinoma revealed molecular subtypes of gastric cancer that partially correspond to histopathologic classification [22]. Diffuse gastric cancer (DGC) is a distinct pathologic and molecular subtype of stomach cancer, defined by both its distinct signet cell ring features, its infiltrative pattern of tissue invasion, and loss of the tumor suppressor *CDH1* (i.e., E-cadherin) that leads to aberrant initiation of the epithelial-to-mesenchymal transition.

We developed a series of new methods that employed barcode linked read analysis to discover cancer rearrangements composed of different SV classes in DGC. We applied these methods to a pair of metastatic diffuse gastric cancers from the same individual. An important concept for this study is that the barcodes and their linked sequences directly represent both the identity and number of single HMW DNA molecules (>50 kb on average). With this information, one can extrapolate the identity of specific DNA molecules that contain SVs. Moreover, the barcode linked reads provide a means to resolve the structure of complex SV events given that genomic contiguity is maintained. Finally, we used the barcode linked sequence data to determine specific haplotype blocks that covered the affected locus. This haplotype information enabled us to infer the parental chromosome origins of the rearrangements. Our analysis identified cancer rearrangements even in the context of having lower fractions of tumor to normal cells. We identified a unique and highly complex *FGFR2* (fibroblast growth factor receptor 2) tandem duplication with a unique structure specific to each metastatic site—this complex rearrangement was not present in the primary tumor. Using an organoid system, we functionally validated the role of *FGFR2* gain-of-function as a potential oncogenic driver associated with metastasis.

## Methods

### Tumor samples and nucleic acid extraction

This study was conducted in compliance with the Helsinki Declaration. The institutional review board at Stanford University School of Medicine approved the study protocol (19071). We obtained a matched set of samples including a gastric primary cancer, two metastases from each ovary, and normal stomach tissue (Additional file 2: Figure S2). These samples were obtained from the Stanford Cancer Institute tissue bank. Based on a formal pathology review, the overall tumor purity of these samples was estimated at less than 40%. Macro-dissection of the tumor samples was performed to increase the tumor DNA fraction in the final extraction. We used the Maxwell 16 FFPE Plus LEV DNA purification kit to extract genomic DNA from the formalin-fixed paraffin-embedded (FFPE) samples and Maxwell 16 Tissue DNA purification to extract DNA from frozen samples according to the manufacturer’s protocol (Promega, Madison, WI, USA). Final DNA concentrations were quantified with the Qubit 2.0 fluorometer (Invitrogen, Carlsbad, CA, USA).

### Linked read library preparation, sequencing, and analysis

For sequencing we used 1 ng of extracted DNA from each of the normal and two ovarian metastatic samples. The Chromium Gel Bead and Library Kit (10X Genomics, Pleasanton, CA, USA) and the Chromium instrument (10X Genomics) were used to prepare the libraries for sequencing. The barcoded libraries were sequenced on an Illumina HiSeq 4000 system. The resulting BCL files were demultiplexed and converted to fastq files using bclprocessor (v2.0.0). The phasing software Long Ranger (v2.0.0) was run to generate a phased call-set of single nucleotide variants (SNVs) and insertion/deletions (indels), and to perform SV discovery.

### Whole genome sequencing

As orthogonal sequencing data for comparison, we conducted conventional WGS on the normal sample and metastatic tumor samples. Whole genome libraries for the normal and metastatic samples were prepared and sequenced with standard TruSeq protocols. The normal and left metastatic sample were sequenced at Illumina (San Diego, CA, USA) on an Illumina 2500 with 100 by 100-bp paired-end reads, and the right metastatic sample was sequenced at Macrogen (Seoul, South Korea) on a HiSeq X with 150 by 150-bp paired-end reads. Sequence reads were aligned to the human genome version GRCh37.1 using the BWA-MEM algorithm of the Burrows-Wheeler Aligner (BWA) v0.7.4 [23] with default parameters. Read mapping and sequencing coverage statistics are listed in Additional file 1: Table S2. The GATK (v3.3) DepthOfCoverage tool was used to calculate coverage metrics [24].

### WGS of FFPE samples

To compensate for the fragmented nature of samples preserved with FFPE, we prepared sequencing libraries for the primary tumor FFPE sample and matched normal FFPE sample using the GemCode Gel Bead and Library Kit (10X Genomics) and the GemCode instrument (10X Genomics). The barcoded libraries were sequenced on an Illumina NextSeq instrument, and the resulting BCL files were demultiplexed and converted to fastq files using bclprocessor (v1.2.0). The aligner function of Long Ranger (v1.2.0) was run to generate aligned bam files. For the FFPE samples, the barcoded nature of the linked reads was used solely to improve alignment of the sequence reads; no phasing was performed for these data as the quality of FFPE samples is not adequate to infer long range haplotypes. Read mapping and sequencing coverage statistics are listed in Additional file 1: Table S2. The GATK (v3.3) DepthOfCoverage tool was used to calculate coverage metrics [24].

### Rearrangement analysis

We used the Long Ranger (v2.0.0) program to identify SV breakpoints. Long Ranger produces a file of SV calls in BEDPE format which reports the start and end positions of the two breakpoints of each SV call. Using these SV calls from our normal and tumor samples, we used a custom Python script to identify the somatic, tumor-specific SVs that represent potential driver events (Additional file 2: Figure S3). Within the script, we implemented the pybedtools package to perform BEDtools [25] intersections of the SV calls in the tumor sample with the SV calls in the normal sample to define somatic events.

Next, we validated the SVs identified from linked read sequencing using SVs identified from independently generated and thus completely orthogonal conventional WGS. Using the conventional WGS data as input, tumor SVs were detected using LumPy and somatic copy number variants (CNVs) were detected using BICseq2 [26, 27]. LumPy was run using the lumpyexpress executable with default parameters, and the output VCF file was parsed to bed format for further processing. For copy number calling, BICseq2 first removes potential biases from the sequencing data (BICseq2-norm v0.2.4) and subsequently calls CNVs from the normalized data (BICseq2-seg v0.7.2). The lambda parameter supplied to BICseq2-seg tunes the smoothness of the resulting CNV profile; a lambda value of 30 was used to call CNVs for the primary tumor and metastatic samples. Amplifications and deletions were called as segments with tumor/normal copy number ratios greater than 1.25 and less than 0.95, respectively.

With the Long Ranger SV output, we generated a file listing the genomic coordinates 5 kb upstream and downstream of the SV breakpoint. Using the results from the LumPy SV caller [28] and the BICseq2 CNV caller [26] on the conventional TruSeq WGS data, we generated another file listing the genomic coordinates 5 kb upstream and downstream of the SV breakpoint. To compare the results between the linked read SVs and conventional WGS SVs, we used pybedtools [25] to identify common overlapping windows per a 5-kb positional proximity.

Finally, we identified those SV events that were located in the vicinity of known and candidate driver genes in gastric cancer. We generated a list of gastric cancer driver genes by selecting the top 10% most frequently mutated genes and the top 10% most frequently copy number variant genes in gastric cancer according to The Cancer Genome Atlas (TCGA) [22]. This ranking generated a total of 3641 unique genes (Additional file 3). We generated 1-Mb windows around SV coordinates and then performed an intersection with the gene coordinates for gastric cancer genes.

### Identifying *FGFR2* copy number using droplet digital PCR

To determine *FGFR2* copy number, we used droplet digital PCR (ddPCR) with a QX200 instrument (Bio-Rad, Hercules, CA, USA) following the manufacturer’s instructions. Briefly, gDNA was first digested by EcoRI-HF (NEB) and cleaned up by AMPure XP beads (Beckman Coulter). Digested gDNA (4 ng) was assayed per 20-μl reaction. The copy number assay primers and probes for *FGFR2* (dHsaCP2500320) and *RPP30* (dHsaCP1000485) reference were obtained from Bio-Rad. After droplet generation, the reaction mixes proceed to thermal cycling as 95 °C × 10 min (1 cycle), 94 °C × 30 s, and 60 °C × 60 s (40 cycles), 98 °C × 10 min (1 cycle), and 12 °C hold. Droplet fluorescence was determined and the QuantaSoft software (Bio-Rad) was used to determine copy number. *FGFR2* copy number was estimated as the ratio of the *FGFR2* and *RPP30* copy number multiplied by two. Each sample was measured in triplicate. As a positive control and standard curve for comparison, we used a gDNA mixture with different ratios of Kato III, a DGC cell line with a known *FGFR2* amplification, and a normal DNA source, NA18507 gDNA (Coriell).

### Structural variant phasing to determine *cis* or *trans* relationships

We developed a bioinformatics process using custom Python and R scripts to analyze barcode information from the linked reads. These scripts provided a graphic representation of barcode information and determined the overlapping haplotypes among individual SV events (Additional file 2: Figure S4). The custom scripts used to process the data are available on GitHub (https://github.com/sgreer77/sv-phasing_linkedreads). For input, we used two Long Ranger result files: (1) the linked read BAM file which provides the mapping location and barcode of each sequence read; (2) the phased VCF file which contains phased variants, haplotypes, and the barcode support for the haplotype assignments. Using the barcode as an identifier for individual DNA molecules (i.e., molecular barcode) was an important component of the analysis. As shown in Additional file 2: Figure S4, the steps of the analysis process are outlined below.

#### Step 1: specify SV events to be phased

The input was the SV BEDPE file containing the SV breakpoints within the proximity of cancer drivers as already described. For a pair of SV calls (*v*_*i*_, *v*_*j*_), breakpoints were defined as in Eq. 1:

1 $$ \begin{array}{c}\hfill {v}_i=\left({x}_i,{y}_i\right)\hfill \\ {}\hfill {v}_j=\left({x}_j,{y}_j\right)\hfill \end{array} $$

Specifically, the variable ${x}_i$ refers to the genomic coordinates proximal to the p arm and the ${y}_i$ refers to the genomic coordinates proximal to the q arm.

#### Step 2: obtain molecular barcodes in windows around breakpoints

For each SV breakpoint, we generated a window segment size, as denoted by the variable *w*, large enough to obtain molecular barcode information from mapped linked reads. The variable $bar\left({r}_i\right)$ refers to the barcode of an individual sequence read. At this step, we obtained the barcodes of all reads that mapped within the window, regardless of any evidence of association with the SV event (Eq. 2):

2 $$ \begin{array}{c}\hfill B\left({x}_i\right)=\left\{ bar\left({r}_i\right)\  s. t.\kern0.5em {r}_i\in \left[{x}_i-\frac{w}{2},\ {x}_i+\frac{w}{2}\ \right]\right\}\hfill \\ {}\hfill B\left({x}_j\right)=\left\{ bar\left({r}_i\right)\  s. t.\kern0.5em {r}_i\in \left[{x}_j-\frac{w}{2},\ {x}_j+\frac{w}{2}\ \right]\right\}\hfill \\ {}\hfill\ B\left({y}_i\right)=\left\{ bar\left({r}_i\right)\  s. t.\kern0.5em {r}_i\in \left[{y}_i-\frac{w}{2},\ {y}_i+\frac{w}{2}\ \right]\right\}\hfill \\ {}\hfill B\left({y}_j\right)=\left\{ bar\left({r}_i\right)\  s. t.\kern0.5em {r}_i\in \left[{y}_j-\frac{w}{2},\ {y}_j+\frac{w}{2}\ \right]\right\}\hfill \end{array} $$

The window size is an adjustable parameter; a 0.1-Mb size provided an adequate number of molecular barcodes for resolving the structure and relationship of SV events. This step was conducted for each sample.

#### Step 3: identify SV-containing molecules

Using the barcodes and their associated reads that mapped to the SV window as described in step 2, we identified the intersecting sets of SV barcodes (Eq. 3):

3 $$ \begin{array}{c}\hfill S\left({x}_i,{y}_i\right) = B\left({x}_i\right)\cap B\left({y}_i\right)\hfill \\ {}\hfill S\left({x}_j,{y}_j\right) = B\left({x}_j\right)\cap B\left({y}_j\right)\hfill \end{array} $$

To identify an SV-containing molecule, the distance between SV breakpoints must be greater than what one would expect to see from the reference genome or represent sequences from different chromosomes. As noted earlier, the aligned sequence data enable us to infer the general molecular size of each molecule per a given partition. We refer to this measurement as the mean molecule length (Additional file 1: Table S3). To verify that the molecules were SV-specific, we performed the same steps using the matched normal linked read data. We expect to obtain few if any SV-specific molecules when using the normal linked read data, as the SV breakpoint regions are not contiguous to each other in the reference genome and therefore should have few if any shared molecular barcodes.

#### Step 4: link/phase SV events

Here, we attempted to phase distinct SV events with respect to one another. We determined if a somatic SV event could be identified from an individual HMW molecule. As noted previously, the molecular barcodes per a given sequence indicate a single droplet partition containing three to five molecules (Additional file 2: Figure S1). Thus, barcodes indicate both the identity and number of DNA molecules within a specific partition. We used the SV-specific molecular barcodes to link different SV events that occurred on the same HMW DNA molecule; this should allow us to link events that are within approximately 50 kb (the average size of a HMW DNA molecule) of one another. For this phasing step, we compared the SV-specific barcodes between each SV event to identify those that were the same (Eq. 4):

4 $$ C\left({v}_i,{v}_j\right) = S\left({x}_i,\ {y}_i\right)\cap S\left({x}_j,\ {y}_j\right) $$

If we observed SV events with the same molecular barcodes, then this was evidence that these events were in *cis* and positioned in the same individual DNA molecule.

Next, we evaluated the SV events that occurred within haplotyped segments (i.e., blocks of phased SNVs or “phase blocks”) of the genome, allowing us to phase events that were more distant from one another (i.e., average phase block size being approximately 1 Mb). Here, we assigned each individual SV event to an existing haplotype scaffold of phased SNVs. For this, we relied on the phased SNVs reported in the Long Ranger VCF files, for both the matched normal and tumor samples. The phased variants of the normal sample were used to define the haplotype structure of the region surrounding each SV breakpoint (Eq. 5); then the phased variants of the tumor sample were used to obtain the supporting molecular barcodes for each allele (Eq. 6):

5 $$ \begin{array}{c}\hfill SNV\left({v}_i\right) = \left\{ snv\  s. t.\kern0.5em  s nv\ \in \left[{x}_i-\frac{w}{2},\ {x}_i+\frac{w}{2}\ \right]\  or\right.\ \hfill \\ {}\hfill \left.\kern9.25em  s nv\ \in \left[{y}_i-\frac{w}{2},\ {y}_i+\frac{w}{2}\ \right]\right\}\hfill \end{array} $$

6 $$ \begin{array}{c}\hfill\ {H}_1\left({v}_i\right) = \left\{ bar(p)\  for\  p\  in\  SNV\left({v}_i\right) s. t.\kern0.5em  hap\left( bar(p)\right)=1\right\}\hfill \\ {}\hfill\ {H}_2\left({v}_i\right) = \left\{ bar(p)\  for\  p\  in\  SNV\left({v}_i\right) s. t.\kern0.5em  hap\left( bar(p)\right)=2\right\}\hfill \end{array} $$

To determine the phase of each SV event, we used the haplotype of the alleles that shared molecular barcodes with the SV-specific molecules (Eq. 7):

7 $$ \begin{array}{c}\hfill\ {R}_1\left({v}_i\right) = {H}_1\left({v}_i\right)\cap S\left({v}_i\right)\hfill \\ {}\hfill\ {R}_2\left({v}_i\right) = {H}_2\left({v}_i\right)\cap S\left({v}_i\right)\hfill \end{array} $$

where *S*(*v*_*i*_) is the set of barcodes that corresponds to *S*(*x*_*i*_, *y*_*i*_).

By assigning each SV event to a haplotype within a phase block, we determined the *cis*/*trans* relationship between the SV events, thus placing them in phase.

### Allele-specific barcode counting from linked reads to determine SV haplotype

To determine the haplotype of an SV event, we performed allele-specific barcode counting [20]. For this, we used a custom Python script in combination with custom R scripts for graphic visualization. First, we used the VCF file of the normal sample to obtain the haplotype assignment of all confidently phased SNVs within a specified region of interest. Our analyses consistently use the normal sample as the source of phasing information since its variants should be phased more accurately than those of the tumor sample. We obtained the number of barcodes assigned to each allele of each phased variant from the matched tumor sample VCF files; thus, we obtained the allele-specific barcode counts. Plotting of these counts depicted whether one or both haplotypes was affected by copy number changes. If only one haplotype was affected, then the identity of the haplotype could be determined.

### SV-specific molecule mapping to resolve SV breakpoint structure

To resolve complex breakpoint structures, we relied on the mapping locations and molecular barcode identities of the linked read sequences, along with the SV-specific molecules for each SV event which were previously determined in our phasing pipeline (Step 3 in Additional file 2: Figure S4). Here, we used a custom Python script to consider a 500-kb window around each SV breakpoint which was then divided into discrete 1-kb windows, i.e., 500 windows were considered for each breakpoint. Based on the linked read BAM file, we quantified the number of times each SV-specific molecular barcode occurred in each 1-kb window. The analysis of this output enabled identification of 1-kb windows where SV breakpoints occurred as those windows with sharp decreases in barcode number. We used a custom R script to plot the mapping locations of reads with each unique molecular barcode, which provides a visualization of the HMW DNA molecule from which each barcode originated. The plot indicates whether each HMW DNA molecule was assigned to haplotype 1 or haplotype 2, as per the assignment of its barcode identifier to SNV alleles in haplotype 1 or haplotype 2.

### De novo assembly of structural rearrangements

We sought to determine whether we could resolve and thus validate the rearranged structure by de novo assembly. We extracted all the sequence reads containing SV-specific barcodes from the linked read fastq files and then used these subset fastq files as input to the Supernova de novo assembly program to generate contig sequences [29]. This assembler has been recently demonstrated to generate full diploid assemblies. We visualized the structures of the resulting contigs by plotting the mapping position of each SV-specific read in the genome versus its mapping position in the contig.

### Gastric organoid cancer modeling in mice and functional analysis

*Cdh1* ^flox/flox^;*Trp53* ^flox/flox^ mice were generated by crossing *Cdh1* ^flox/flox^ mice, obtained from Jackson Laboratory, and *Trp53* ^flox/flox^ mice, kindly provided by Dr. Anton Berns [30]. NOD.Cg-*Prkdc* ^*scid*^ *Il2rg* ^*tm1Sug*^/JicTac (NOG) mice were obtained from Taconic Biosciences, Inc. The Stanford University Administrative Panel on Laboratory Animal Care approved all animal experimental protocols. We dissected stomachs from neonatal mice (age P4–7) and washed them in cold PBS to remove all luminal contents. We extensively minced each entire neonatal stomach and embedded the minced tissues in a 3D collagen gel using a double-dish culture system as previously described [31]. To maintain the organoids, we applied fresh media (F12, 20% FBS, Gentamicin 50 μg/mL) every week.

We obtained the retroviral construct pBabe-puro-*FGFR2* from Dr. Channing Der [32]. Retroviral plasmids were cotransfected with pCL-Eco into 293 T cells by Lipofectamine2000 (Invitrogen). Retroviral supernatants were collected 48 and 72 h post-transfection and concentrated by PEG-it virus precipitation solution (System Biosciences). We determined the virus titer by infection of NIH/3T3 cells and FACS analysis of GFP-positive cells 48 h post-infection. We used the adenovirus AdCre-GFP and Ad-Fc to infect the organoid cultures at day 0 by applying directly to the surface of collagen containing primary tissue. Retroviral particles were incubated with pellets of dissociated primary organoids at room temperature for 45 minutes before serial replating into 3D collagen gel.

We fixed samples with 4% paraformaldehyde overnight, then paraffin-embedded and sectioned them. We stained deparaffinized sections with H&E for initial histology analysis. For further immunohistochemistry analysis, we used antibodies for the following proteins: PCNA (1: 300; Invitrogen), E-cadherin (1: 300; BD Biosciences Pharmagen), p53 (1: 100; Santa Cruz), and FGFR2 (1:300; Sigma).

Gastric cells were collected from collagen gel by disaggregation with collagenase IV (Worthington). For transplantation, 400,000 cells per mouse flank were mixed with Matrigel (50% Matrigel, 10% FBS, 40% F12, 100 μl of Matrigel mixture for one mouse) and injected into NOG mice. Mice were sacrificed after day 50 and we dissected the tumors and examined tumor sections with H&E staining. *P* values were determined using a two-tailed Student’s *t*-test assuming unequal variances. A *p* value of 0.05 was considered significant.

## Results

As a proof-of-concept study, we applied linked read WGS with barcodes to the gastric tumors from an individual with recurrent metastatic cancer. These tumors came from a surgical resection of metastatic sites located in the right and left ovary (Additional file 2: Figure S2). Both metastases were present at the time of the surgical procedure. Histopathology confirmed that all three sites (i.e., right metastasis, left metastasis, and primary gastric tumor) were diffuse gastric cancer. This represents clinical confirmation that the metastases originated from the primary gastric tumor.

### Linked read sequencing of gastric cancer metastases

Using genomic DNA from the two metastatic sites as well as the matched normal tissue, we performed linked read WGS (Additional file 2: Figure S1). In addition to linked read sequencing of the metastatic samples, we also conducted a conventional WGS analysis as an orthogonal and completely independent validation dataset. The primary tumor tissue was an FFPE sample and thus the DNA quality was inadequate for linked read sequencing. However, conventional WGS was performed for this primary tumor sample (Additional file 1: Table S2).

The linked read method uses massively parallel partitioning of HMW DNA alongside droplet barcoding to create haplotypes of variants including SNVs and indels [20]. The mean sequencing coverage achieved using linked read sequencing for the normal, right metastatic, and left metastatic samples was 36.0, 20.1, and 35.4, respectively (Additional file 1: Table S2). The largest molecule lengths and the longest phase blocks were obtained in the normal sample, where the mean molecule length was ~51 kb and the N50 phase block size was 1.4 Mb. In contrast, the smallest mean molecule length and N50 phase block size were achieved in the right metastasis, at 45 kb and 0.63 Mb, respectively. These results demonstrated that linked read sequencing provided long-range genomic contiguity on the scale of tens of kilobases, compared with conventional WGS. The N50 and molecule length differences were a result of variation in size of the DNA and the extent of fragmentation. This factor likely contributed to the greater proportion of SNVs phased in the normal sample (99%) compared with either tumor sample, where 98.2 and 98.9% of SNPs were phased in the right and left metastases, respectively (Additional file 1: Table S3).

### Identification of cancer SVs from linked reads

From our linked read analysis, we identified a series of somatic SV events; seven SVs occurred in the right metastasis and 17 events occurred in the left metastasis (Additional file 1: Table S4). The right metastasis harbored three deletions, one duplication, one inversion, one translocation, and one nonspecific distal event. In contrast, the left metastasis harbored eight deletions, three duplications, two inversions, two translocations, and two distal events. Two deletion events and one translocation event were common to both metastatic tumors. The shared translocation was an inter-chromosomal event between chromosome 11q13.5 and 19p13.12, which putatively impacts the chromatin-remodeling gene *RSF1* located at chromosome 11q14.1. Previous studies have shown a correlation between *RSF1* upregulation and tumor aggressiveness in multiple cancer types [33, 34], potentially by causing chromosomal instability [35].

The chromosomal region harboring *FGFR2* was duplicated in both metastases. However, the SV analysis revealed that the breakpoints of the amplification event differed between the left and right site (Additional file 1: Table S4). Furthermore, additional SV events were detected in the region surrounding the *FGFR2* amplification, with a series of unique breakpoints specific to each metastasis. This indicated that a potentially complex rearrangement had occurred in the *FGFR2* locus, and suggested an independent occurrence of the somatic SVs between the two metastatic sites.

All samples were subject to a separate, independent sequencing analysis with conventional WGS. We used these data to independently confirm the SV calls from the phased sequencing and barcode linked reads. For analysis of the conventional WGS data, we used both an SV caller and a CNV caller, Lumpy [28] and BICseq2, respectively. Lumpy identified 485 somatic SVs in the right metastasis, five of which were shared with the seven Long Ranger SV calls. Similarly, Lumpy identified 493 somatic SVs in the left metastasis, seven of which were shared with the 17 Long Ranger SV calls (Additional file 1: Tables S4 and S5). Long Ranger reported fewer SVs than LumPy because Long Ranger specializes in detecting larger SV events (i.e., the smallest SV we detected with Long Ranger was ~30 kb). In addition, Long Ranger sets stringent filtering parameters such as ignoring those SVs that occur within or near repetitive genomic regions. The variation between Lumpy and Long Ranger is due to different algorithms, and it has previously been shown that there is generally very little overlap among the results of different SV callers [36].

We performed CNV segmentation on the conventional WGS data using BICseq2 (“Methods”). BICseq2 identified 42 somatic CNVs in the right metastasis (29 amplifications and 13 deletions). Only 16 somatic CNVs were detected by BICseq2 in the left metastasis (two amplifications and 14 deletions; Additional file 1: Table S4; Additional file 2: Figure S5).

### Identification of *FGFR2* amplification*s* in both metastatic samples

As described, our CNV and SV analyses detected an amplification of the region surrounding *FGFR2* in both metastases; this result came from the orthogonal analyses of the conventional and linked read WGS data. However, no amplification was detected in the primary tumor from the conventional WGS data. As added confirmation of our copy number results, we used a highly sensitive ddPCR assay to assess the *FGFR2* status of the primary tumor, ovarian metastases, and matched normal gastric tissue. The ddPCR CNV assay detected the *FGFR2* amplification in both metastases but not in the primary tumor or matched normal sample (Additional file 2: Figure S6). According to the ddPCR analysis, *FGFR2* copy number (CN = ~9) was higher in the right metastasis compared with the left metastasis (CN = ~6), which is concordant with what was observed for the WGS CNV results.

### *FGFR2* rearrangement structure in the metastases

**Fig. 1:**
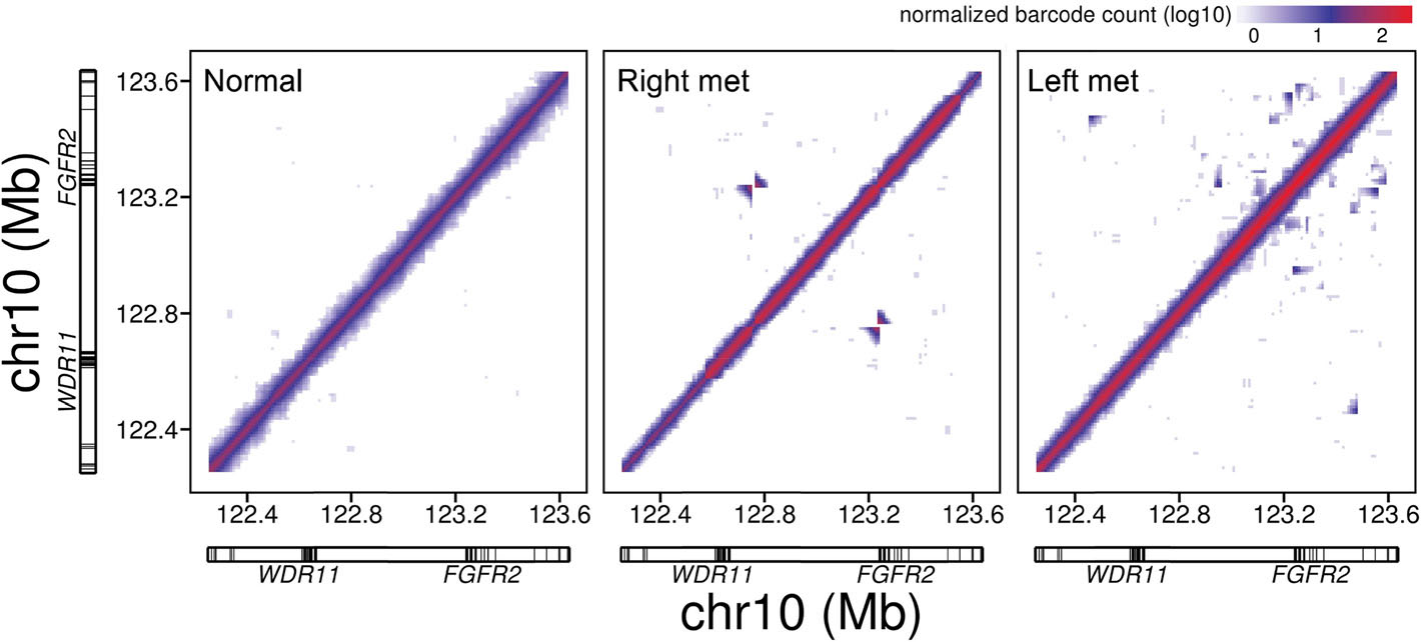
Barcode overlap plots of the genomic region surrounding the proto-oncogene *FGFR2*. The level of barcode sharing between 10-kb windows in a 1.4-Mb genomic region including *FGFR2* was determined for the normal sample and the right and left metastatic samples. The highest level of overlap (*red*) is expected along the diagonal, while off-diagonal signals (*red* or *blue*) indicate the presence of structural variants

Many cancer amplifications are related to tandem duplications. We used linked reads and molecular barcodes to determine the nature of the amplifications and the structures of the underlying duplications. With the linked read WGS data from each metastatic site, our analysis identified a number of unique SV events in the chromosomal region from 10q23.31 to 10q26.13 that harbors the *FGFR2* gene, a gastric cancer driver (Additional file 1: Tables S4 and S6). Moreover, the SV breakpoints in this region were unique to each metastasis, suggesting that rearrangement of this region had occurred independently. The complexity and differences among the samples for this chromosomal region are clearly displayed in plots of barcode overlap. Off-diagonal signals represented SV events (Fig. 1; Additional file 2: Figure S7). The patterns between the left and right metastasis are very distinct with little overlap.

As we previously reported, we demonstrated and validated that one can generate cancer genome megabase-scale haplotypes from primary tumors and these haplotypes enable one to reconstruct somatic SVs and rearrangements that extend over megabases [20]. We improved on this process for linking haplotypes and applied it to better characterize the rearrangement that led to the *FGFR2* gene amplification. First, we sought to determine the haplotypes encompassing the SV events. For each metastasis, this analysis involved phasing the SV events and determining if the SVs were either in *cis* or in *trans* with one another. This method takes advantage of the long-range barcode information and haplotype variants associated with the linked read data (“Methods”). Our method and related steps are outlined in Additional file 2: Figure S4.

In the right metastasis, we evaluated three SV events: a duplication, a deletion, and an inversion (Table 1; Additional file 1: Table S7). SV breakpoints were denoted by a start and end position; we used this interval to define larger, 100-kb windows around each breakpoint from which to obtain molecular barcodes. The 100-kb windows around the start and end breakpoints of the duplication contained a total of 1315 and 1287 unique molecular barcodes derived from the linked reads, respectively. Of these “breakpoint-specific” barcodes, 119 were shared between the breakpoints and thus represented the molecular barcodes of the SV-specific molecules of this event. Using this method, we also obtained 158 SV-specific molecules for the deletion event and 313 for the inversion event. The SV-specific barcodes and inferred molecules were used to phase SV events relative to each other.

**Table 1:** SV phasing results for SVs in the right metastasis in the region surrounding *FGFR2*

SV ID	SV breakpoints	Number of unique molecules in breakpoint window	SV breakpoint-spanning molecules	Somatic phase block coordinates	Number of molecules assigned to haplotype	Molecule support for haplotype assignment	
						Hap1	Hap2
DUP	92790689	1315	119	91045792 – 95201175 + 122189628 – 125868860	92	92 / 92 (1.0)	0 / 92 (0)
	123553942	1287					
DEL	93321593	1474	158		80	80 / 80 (1.0)	0 / 80 (0)
	122582235	1582					
INV	122756078	2081	313		201	200 / 201 (0.99)	1 / 201 (0.01)
	123240170	2486					

We determined whether any of the SV events occurred on the same DNA molecules, thus indicating that the individual SVs were in *cis*. As described, we determined that the average molecule size was approximately 50 kb for these samples. For any pair of SVs to be in *cis* and also present in the same DNA molecule, we would anticipate that common barcodes would be present. We refer to this subset as molecule barcode overlaps and SV-specific molecules. In the case of the right metastasis, no molecules were shared between events, indicating that either the SV events were too distant from one another to be detected from the same HMW molecule (average size ~50 kb) or the SVs occurred in *trans*.

To phase SVs that were in genomic positions too far apart to be phased based on molecular barcode overlap, we relied on the haplotype information. First, we assigned each SV to a haplotype block based on overlap between SV-specific molecules and the phased heterozygous SNVs. Both the SNVs and SVs are denoted by barcodes. Using both the barcode and haplotypes to which a given set of SNVs are assigned, one can identify those SV barcodes with a matching SNV barcode. These “overlapping” barcodes determine the haplotype block encompassing the SV.

In the case of the right metastasis, we extended our analysis to link distant haplotypes covering the start and end breakpoints of individual events. We denote these breakpoints as DUP (duplication), DEL (deletion), and INV (inversion) (Additional file 1: Table S7). Of the 119 SV-specific molecules for the duplication event, 92 could be assigned to one or the other haplotype using barcode comparisons; all of these molecules (92/92) were assigned to haplotype 1, and none were assigned to haplotype 2. The same trend was observed for all three SV events in this region of the right metastasis with all of them being assigned to haplotype 1. Thus, we concluded that all of these SV events were in *cis* with one another, existing on the same haplotype.

We performed this same SV phasing analysis for the left metastasis. For the *FGFR2* locus, there were five discrete SV events: two duplications, two deletions, and an inversion (Table 2; Additional file 1: Table S7). One of the duplication events (DUP1) was identified by Lumpy but not by Long Ranger, and was included based on its occurrence within our region of interest, i.e., proximal to *FGFR2*. For each event, we were able to identify between 49 and 83 SV-specific barcodes. A duplication event (DUP2) and a deletion event (DEL1) shared 28 molecular barcodes, indicating 28 HMW DNA molecules spanned both of these events. These two events were in *cis* with one another. The inversion event and a deletion event (DEL2) shared two SV-specific molecules, indicating a potential *cis* relationship between these SVs.

**Table 2:** SV phasing results for SVs in the left metastasis in the region surrounding *FGFR2*

SV ID	SV breakpoints	Number of unique molecules in breakpoint window	SV breakpoint-spanning molecules	Number of molecules shared between SVs	Phase block coordinates	Number of molecules assigned to haplotype	Molecule support for haplotype assignment	
							Hap1	Hap2
DUP1	122465822	2176	49	0	122189628 – 125868860	42	41/42 (0.98)	1/42 (0.02)
	123486940	3542						
DUP2	122946842	3051	81	28		60	59/60 (0.98)	1/60 (0.02)
	123782540	2588						
DEL1	122959061	3198	73			21	21/21 (1.0)	0/21 (0)
	123242792	4216						
INV	123237230	4230	83	2		58	37/58 (0.64)	21/58 (0.36)
	123563811	2635						
DEL2	123555077	2721	71			63	58/63 (0.92)	5/63 (0.08)
	123709721	2423						

For the left metastasis, the other SV events did not occur on the same HMW DNA molecule due to either distance or a *trans* relationship; therefore, we assigned the SVs to haplotypes. All of the SV breakpoints occurred on one haplotype relative to one another. In all cases, the majority of the SV-specific molecules belonged to haplotype 1, indicating a *cis* relationship for all of these SV events. Interestingly, the inversion event showed relatively high identity with both haplotypes, with 37 from a total of 58 and 21 from a total 58 SV-specific molecules being assigned to haplotype 1 and haplotype 2, respectively, indicating that a rearrangement event at this genomic locus affected both haplotypes. Using this new approach, we assigned a haplotype to the duplication event that was not called by the Long Ranger software. This result indicates that our SV haplotyping method provides inferences that are not immediately observed with SV calling from linked read data.

### Allele-specific barcode counts confirm the haplotype of the rearrangement

Our analysis of the WGS linked reads generated genome-wide phased heterozygous variants and barcodes of all associated reads that have a variant allele assigned to a given haplotype. We leveraged these two major features to verify the haplotype segment covering the *FGFR2* rearrangement. First, the barcode count for each allele of a variant provided allele-specific copy number information. Second, each haplotype has one of two alleles for any given SNV position and the representation of each allele can be quantified based upon barcode counts. By using and comparing these two features, we determined the haplotype composition of the *FGFR2* rearrangement. Comparing the tumor haplotypes to the germline haplotypes from the normal tissue, we were able to confidently assign common haplotypes.

**Fig. 2:**
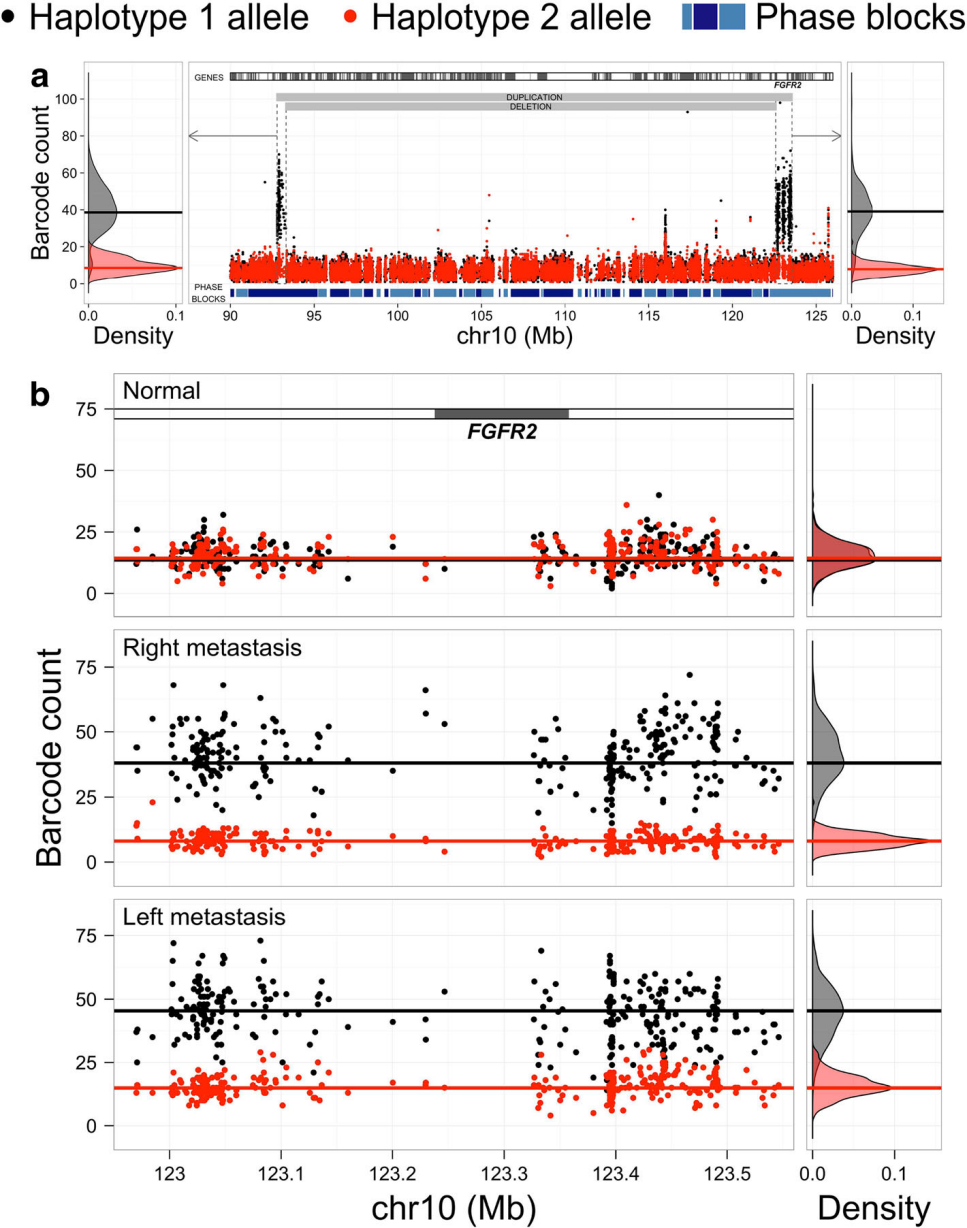
Allele-specific barcode counts. **a** For the right metastasis, the number of barcodes associated with each allele of all phased heterozygous variants is shown for a 36-Mb genomic region including *FGFR2*. The allelic barcode counts are colored in *black* and *red* to denote belonging to haplotype 1 or haplotype 2 within each phase block. The locations of the duplication and deletion events, as identified by Long Ranger, are indicated. The barcode count densities are plotted for each amplified region before and after the deletion event (regions denoted by *dashed rectangles*). **b** Allele-specific barcode counts for each phased allele in the tumor-amplified region of *FGFR2*, using the normal sample to define allelic assignment to haplotype 1 (*black*) or haplotype 2 (*red*). The same haplotype (haplotype 1; *black*) is amplified in both metastases

To confirm the *cis* relationship of the duplication and deletion events in the right metastasis, we leveraged the barcode count data from all phased SNVs across the 90 to 126 Mb region of chromosome 10. These phased SNVs defined the haplotype blocks encompassing the entirety of the genomic segment containing these SV events. Overall, the amplification consisted of a series of duplicated segments but also contained an internal deletion event. Based on examining the barcode information by haplotype, we confirmed that these events both occur on the same copy of chromosome 10 (Fig. 2a). The alleles from only one haplotype demonstrated an increase in barcode counts as shown in Fig. 2a. Haplotype 1, shown as black dots and the grey density plot on the side, showed a significant increase in barcode counts across the genome amplification segment compared to haplotype 2, shown as red dots and density plot. In comparison, the normal diploid genome showed overlap of the allele barcode counts for either haplotype, as one would expect for a normal diploid genome.

For the left metastasis, the allele-specific barcode counts also supported the occurrence of two distinct duplication events on the same haplotype (Additional file 2: Figure S8). It was evident that the barcodes of haplotype 1 were preferentially elevated in the genomic region containing two distinct duplication events, but barcode counts were highest where the two events overlapped one another. Again, this evidence supported our conclusion that the duplication/amplification events were restricted to a single haplotype in this metastasis.

### Comparative allele-specific barcode counting reveals a common rearranged haplotype

Given the distinctive structural differences between the two metastases at the *FGFR2* locus, we determined whether both metastases had the same haplotype involved in the *FGFR2* amplification. As just described, our phasing analysis revealed that the SV events in the chromosomal region 10q23.31 to 10q26.13, encompassing *FGFR2*, were generally restricted to one haplotype in each metastasis. To conduct this comparison, we examined the *FGFR2* locus from 10q23.31 to 10q26.13 and focused our analysis on the common segment where the amplification/duplication was observed in both metastases. We made comparisons of both metastases’ haplotypes with the germline haplotype structure (e.g., same phased SNV genotypes) as determined from the normal tissue. As depicted in Fig. 2b, the allele-specific barcode counts showed that the same haplotype was amplified in both metastases (haplotype 1; Fig. 2b). As we noted, we made this haplotype assignment with high confidence based on the specific genotypes assigned to haplotype 1 versus haplotype 2.

### SV-specific molecule mapping to resolve SV breakpoint structure

**Fig. 3:**
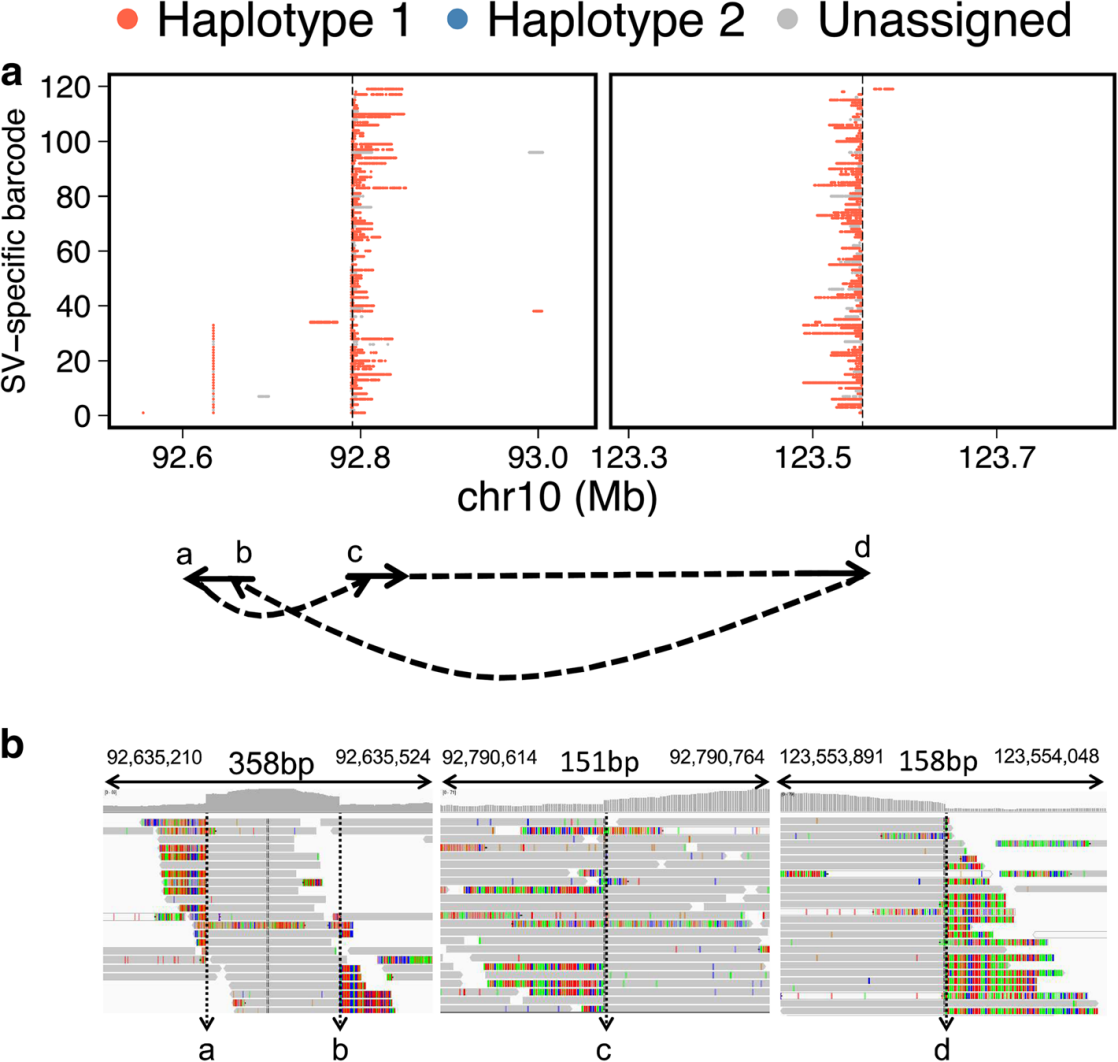
Complex breakpoint resolution using molecular barcode mapping. **a** The SV-specific molecules for breakpoint 1 and breakpoint 2 of the duplication SV in the right metastasis are plotted according to the mapping location of molecular barcoded reads. Each row of the plot represents one SV-specific molecule, depicting how each SV-specific molecule spans the SV breakpoint. Molecular breakpoints are denoted with a, b, c, and d, and the *arrow structure* indicates breakpoint connection and directionality. **b** IGV plots of the molecular breakpoints display soft-clip evidence of the breakpoints

**Fig. 4:**
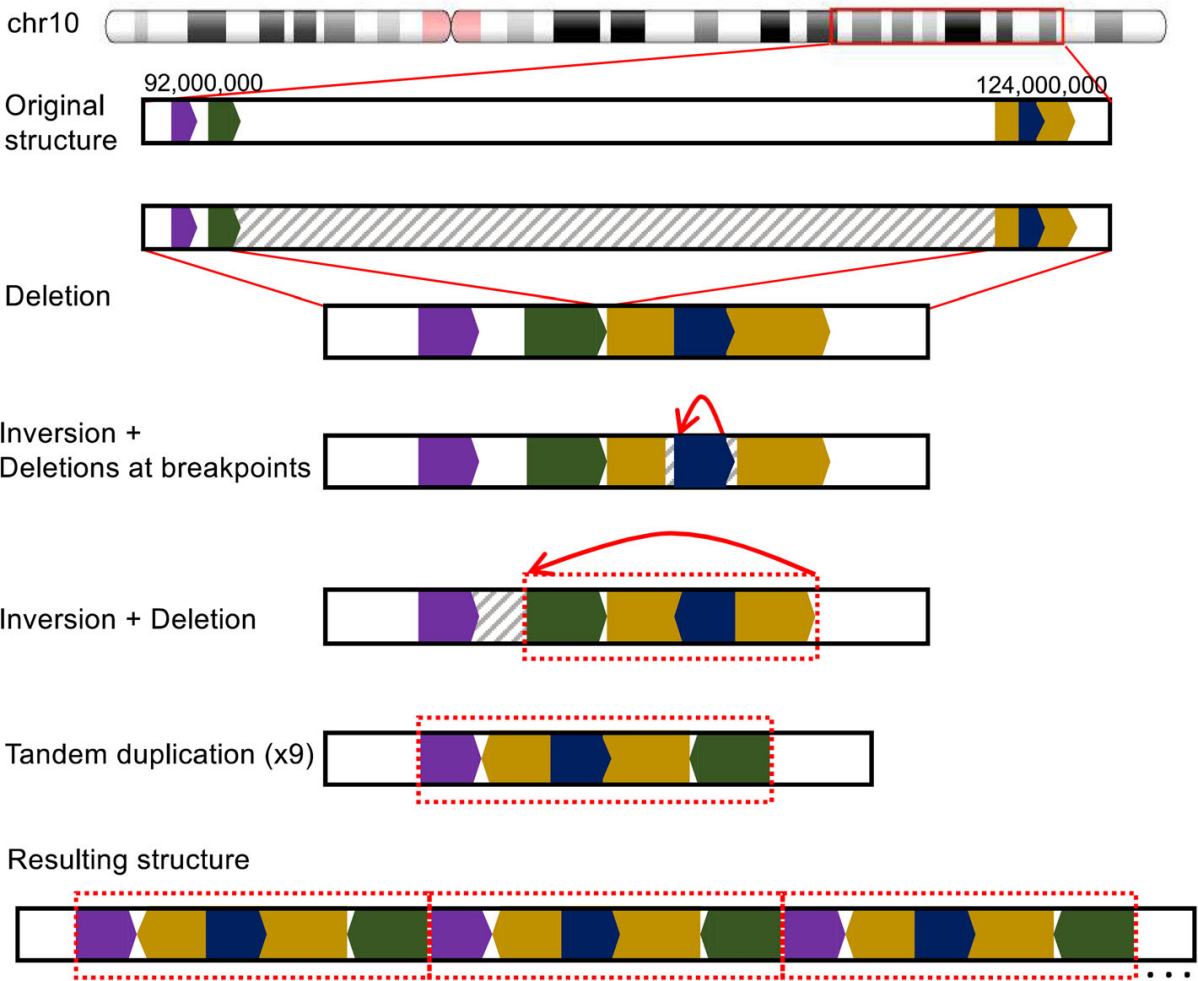
Putative structural rearrangement of the *FGFR2* genomic region in the right metastasis. Barcode and read-based evidence indicate the likely occurrence of events was a 30-Mb deletion event with a nearby inversion event, and an inversion event with a deletion at the boundary; the resulting rearrangement then underwent an approximately ninefold tandem duplication. Barcode analysis indicates that all of these events are in *cis* with one another and thus occurred on only one copy of chromosome 10

To resolve the structure of complex SV breakpoints, we leveraged the molecular barcodes of linked read sequencing to map the genomic coordinates of the original HMW DNA molecules (Fig. 3a). Using this method, we determined the structure of the duplication breakpoint in the right metastasis. Our analysis indicated that the genomic region between breakpoint ‘c’ and breakpoint ‘d’ (Fig. 3a) was inverted and shifted such that breakpoint ‘d’ connected to breakpoint ‘b’, and breakpoint ‘a’ connected to breakpoint ‘c’. Thus, we were able to resolve the breakpoint structure of the tandem duplication; this structure was supported by split-read and read-pair evidence from conventional WGS sequencing data (Fig. 3b). The same HMW molecule reconstruction was performed for the other SV events of the right metastasis. The deletion had a simple breakpoint structure with molecules spanning the junction (Additional file 2: Figure S9). In contrast, the inversion SV was more complex, with deletions at each of the inverted breakpoints (Additional file 2: Figure S10). Figure 4 provides a putative, complete structure for the *FGRF2* rearrangement characterized in the right metastasis. We illustrate how the different component SVs such as the genomic deletions and inversion led to a common genomic motif that underwent duplication.

By mapping the breakpoint of the inversion event detected in the left metastasis, we observed that two distinct rearrangement events occurred on each haplotype in close proximity to one another (Additional file 2: Figure S11). The inversion event identified by Long Ranger belonged to haplotype 1 only.

### De novo local assembly of the *FGFR2* rearrangement

To validate the putative rearrangement structure of the *FGFR2* region, we performed a de novo assembly using all of the reads labeled with SV-specific barcodes (i.e., SV-specific reads). For the right metastatic sample, from ~400,000 reads, we generated 35 contigs with a contig N50 value of 33 kb (Additional file 1: Table S8). Of these contigs, six aligned to multiple positions in the genome (i.e., indicating potential split mapping across a breakpoint) and two of these had contig sizes greater than the assembly N50 value. These two contigs contained all of the breakpoints in the putative rearrangement; contig 1 was ~40 kb and crossed the duplication breakpoint, while contig 2 was ~150 kb and crossed the deletion and inversion breakpoints (Additional file 2: Figure S12). A comparison of where the SV-specific reads aligned to the contigs versus where they aligned to the genome revealed the structure of the contigs that supported our proposed putative rearrangement (Additional file 2: Figure S12). For the left metastasis, we used ~300,000 reads that fulfilled the SV criteria and the assembly revealed 53 contigs with an N50 of ~9 kb (Additional file 1: Table S8). The largest was 12 kb and aligned to the reference without evidence of breaks. Nine contigs aligned to multiple positions in the genome and only one of these had a contig size greater than the assembly N50 value. This contig incorporated the breakpoint of the SV represented as DUP2 (Table 2), thus providing additional validation of our analysis method.

### *FGFR2* gain-of-function in gastric organoids leads to gastric cancer and metastasis

To functionally validate the potential role of *FGFR2* in metastatic diffuse gastric cancer, we developed an in vitro gastric organoid culture system to model candidate driver combinations from the primary tumor and metastasis. Previously, we reported long-term in vitro primary intestinal organoid culture utilizing an air–liquid interface, incorporating both epithelial and mesenchymal elements and preserving multilineage differentiation, intestinal stem cells, and the endogenous Wnt- and Notch-dependent stem cell niche [31, 37].

Using this approach, gastric organoids were established from neonatal murine tissue (Additional file 2: Figure S13a,b). After a 50-day maintenance period, the gastric organoids were checked for terminal, multilineage differentiation. This was confirmed by the presence of H^+^/K^+^ ATPase-positive and mucin-producing epithelial cells (Additional file 2: Figure S13c–e). Immunofluorescence for proliferating cell nuclear antigen (PCNA) identified active mitosis (Additional file 2: Figure S13g). The gastric organoids were genetically tractable and easily engineered by adenovirus or retroviral infection (Additional file 2: Figure S13i, j).

Since the patient’s metastatic tumors harbored *CDH1* and *TP53* mutations, primary gastric organoids were established from *Cdh1* ^fl/fl^, *Trp53* ^fl/fl^ neonatal mouse stomach. These gastric organoids were infected with adenovirus Cre-GFP to induce recombination and deletion of *Cdh1* and *Trp53*, thus modeling the key driver alterations common to both the primary and metastatic tumors. The genomic deletion of *Cdh1* and *Trp53* was confirmed by PCR.

To model the effect of the *FGFR2* amplification event in the two ovarian metastases, the *Cdh1* ^-/-^;*Trp53* ^-/-^ gastric organoids were further infected with an *FGFR2* human retrovirus. We confirmed the FGFR2 receptor overexpression by immunofluorescence (Additional file 2: Figure S14a). Gastric organoids with the *Cdh1* ^-/-^;*Trp53* ^-/-^; *FGFR2* cDNA demonstrated large, irregular nuclei and occasional signet rings consistent with the histological features of DGC (Additional file 2: Figure S14b).

**Fig. 5:**
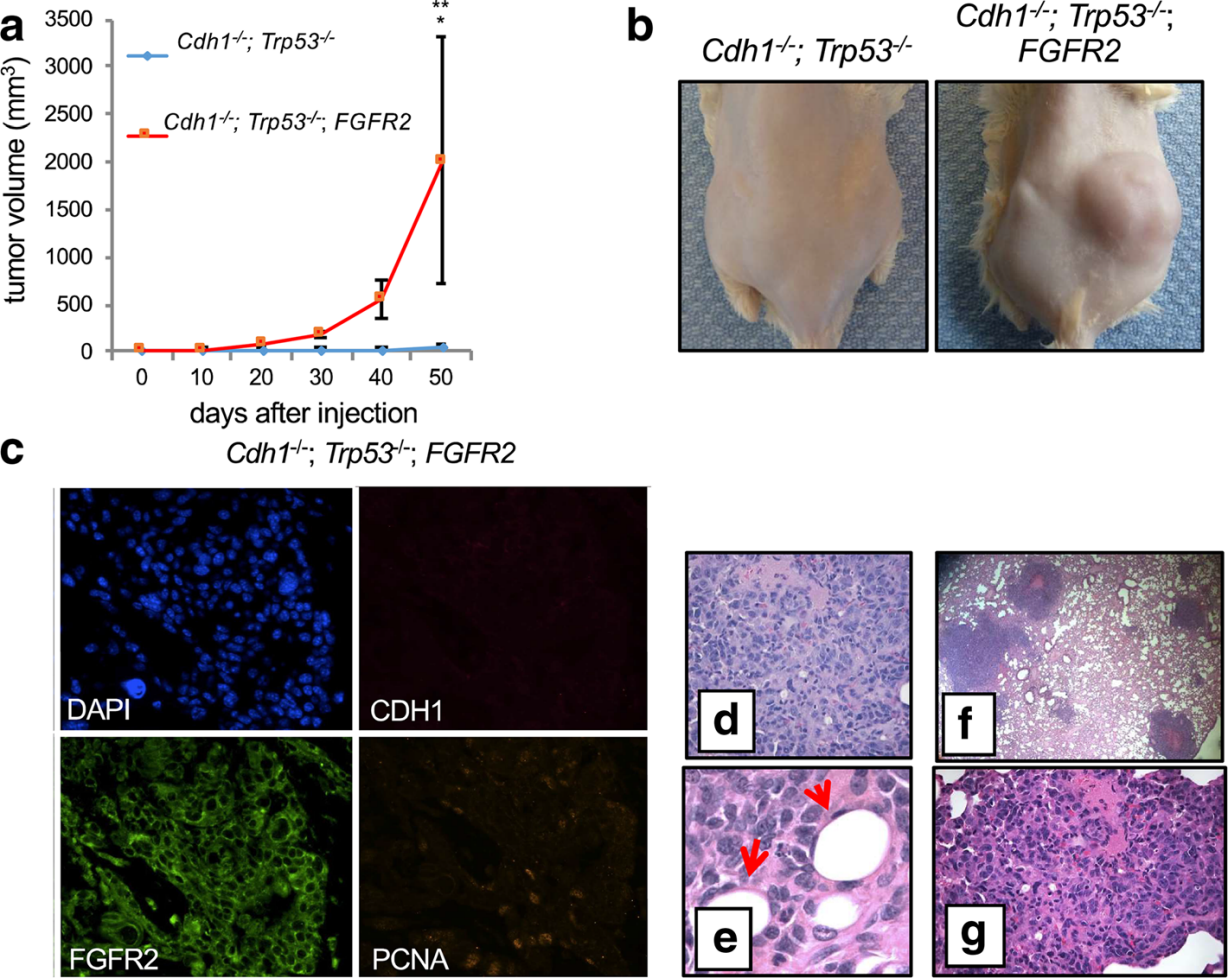
Gastric organoid tumor model. Gastric organoids with the indicated genotypes are shown. **a** Tumor volumes were measured over time post-injection. Gastric organoids were dissociated and subcutaneously injected into the flanks of NOG mice. *Cdh1* ^-/-^;*Trp53* ^-/-^ is shown in *blue*, and *Cdh1* ^-/-^;*Trp53* ^-/-^;*FGFR2* is shown in *red. Error bars* represent SEM, and asterisks indicate *p* < 0.04. **b** Images indicate tumor growth at 50 days post-injection. **c** Overexpression of *FGFR2* was confirmed in the tumor derived from *Cdh1* ^-/-^;*Trp53* ^-/-^;*FGFR2* organoids. **d**–**e** Histological analysis of the *Cdh1*-/-;*Trp53*-/-;*FGFR2* tumors confirms the presence of poorly differentiated adenocarcinoma with signet ring as indicated by *arrows*. **f**, **g** After flank injections with dissociated organoids, histological analysis of murine lungs after 50 days revealed metastatic gastric adenocarcinoma with signet ring features at low (**f**) and high (**g**) magnification

The transformed *Cdh1* ^*-/-*^ *;Trp53* ^*-/-*^ *;FGFR2* cDNA organoids were disaggregated and injected subcutaneously into the flanks of immunodeficient NOG mice. The *Cdh1* ^*-/-*^ *;Trp53* ^*-/-*^ *;FGFR2* organoid xenografts showed rapid development of primary gastric tumors (Fig. 5a, b). In stark contrast, *Cdh1* ^*-/-*^ *;Trp53* ^*-/-*^ mice had no apparent tumors by day 50 (Fig. 5a, b). Gastric organoids with *Cdh1* ^*-/-*^ *;Trp53* ^*-/-*^ *;FGFR2* cDNA exhibited a poorly differentiated adenocarcinoma histology with signet ring features (Fig. 5d, e). Immunofluorescence analysis showed loss of *Cdh1* expression and the specific overexpression of FGFR2 in respective subcutaneous organoid tumors transformed with *FGFR2* retrovirus (Fig. 5c). Evaluation for distant disease confirmed the presence of pulmonary metastases in the lungs of NOG mice harboring subcutaneous *Cdh1* ^*-/-*^ *;Trp53* ^*-/-*^ *;FGFR2* tumors. Similar to primary subcutaneous tumors, histological analysis of the metastatic tumors confirmed poorly differentiated adenocarcinoma with signet ring features (Fig. 5f, g).

## Discussion

Determining the structure of cancer rearrangements remains a difficult task. Short-read, conventional WGS remains the most widely used method for identifying somatic rearrangements in tumors but results are far from perfect and complete resolution of complex genomic structures is near impossible for large events, due to insufficient read coverage at breakpoints and loss of long-range genomic contiguity. For this study, we successfully applied a novel sequencing approach that generates linked read sequences with barcodes to analyze metastatic diffuse gastric tumors obtained from surgical resections.

This sequencing technology addresses many of the challenges associated with SV detection and resolution. Linked read sequencing retains long-range genomic information by enabling sequence reads to be derived from HMW DNA molecules, on the order of 50 kb, without the loss of long-range contiguity due to fragmentation during library preparation. Given that genomic contiguity is maintained, we applied this technology to detect SVs. Moreover, we developed a method that leverages barcoded reads to phase SVs relative to one another (i.e., determine *cis*/*trans* relationships between SVs) and to resolve complex breakpoints from primary tumor samples obtained by surgical resection as opposed to cancer cell lines.

To demonstrate the utility of this approach for sequencing cancer genomes from tissue samples (as opposed to cancer cell lines), we analyzed two synchronously occurring metastatic diffuse gastric cancers that were present in the same individual. This type of gastric carcinoma has a worse prognosis compared to the other molecular subtypes and extremely restricted treatment options [38, 39]. Patients with diffuse gastric cancer invariably succumb to tumor metastasis. Despite its lethality, we know very little about the underlying genetics and biology of DGC metastatic progression—our results indicate that metastatic drivers may be absent in the primary tumor [40]. In addition, our study is unique given that there are few if any genomic or WGS results from DGC metastases [41].

Using this new sequencing approach, we identified a complex rearrangement of the *FGFR2* locus, located on the q arm of chromosome 10. In both metastases, these SV events resulted in amplification of *FGFR2*, as reported by barcode counts from linked read sequencing and CNV calling from conventional WGS data. *FGFR2* is a transmembrane receptor that acts as part of a key signal transduction pathway regulating tissue repair and embryonic development among a host of other functions [42]. *FGFR2* amplification occurs in 5–10% of gastric cancers, with an association to poor diagnosis and tumor metastasis [43, 44]. Preclinical models have shown that *FGFR2* signaling activation due to *FGFR2* amplification is an essential driver for a subset of gastric cancers [45, 46]. In addition, treatment of gastric cell lines with *FGFR2*-specific small molecule inhibitors or short hairpin RNAs (shRNAs) leads to potent growth inhibition [47], suggesting a functional role for *FGFR2* amplification in DGC.

Interestingly, *FGFR2* amplification was not observed in the primary tumor sample of our study patient, and the SV breakpoints of the *FGFR2* region rearrangement were unique in the right and left metastases. This suggested that amplification of *FGFR2* occurred independently in each metastasis, underscoring a potential association of *FGFR2* amplification to metastasis in DGC. Leveraging the long-range genomic information using the molecular barcodes from linked reads, we determined the identity of the HMW DNA molecules and used this information to resolve how the various somatic SVs contributed to a tandem duplication that increased the *FGFR2* copy number. The putative structure for the rearranged region in the right metastasis included a 30-Mb deletion, an inversion with deletions at each of its breakpoints, a subsequent inversion with an associated deletion, and finally a tandem duplication. This structure would have been extremely difficult to resolve, with much less supporting evidence, without the long-range barcode information of linked read sequencing.

We were able to validate the putative rearrangement structure of the *FGFR2* region using de novo assembly to generate long contiguous sequences (Additional file 2: Figure S12). Other useful validation approaches could include long-read sequencing (e.g., Pacific Biosciences, Oxford Nanopore) or optical mapping (e.g., BioNano). However, we did not perform these technologies within the scope of this study, in part due to the high sample input requirements, the higher error rates that may affect SNVs used in haplotyping analysis, the requirement for greater sequencing coverage given the low tumor fraction, and the higher sequencing cost for whole genome analyses (Additional file 1: Table S1).

We provided additional results supporting the potential role of *FGFR2* as an oncogenic driver in DGC. The results from an in vitro organoid mouse model demonstrated that *Cdh1* ^-/-^; *Trp53* ^-/-^ organoids did not form tumors when injected into NOG mice, while *Cdh1* ^-/-^; *Trp53* ^-/-^ organoids with *FGFR2* overexpression did promote tumor growth (Fig. 5). What’s more, the organoid-derived tumors with *FGFR2* overexpression had histologic features of gastric cancer and caused metastases to the lung.

## Conclusions

As genomic analysis plays an increasingly prominent role in advanced cancer patients, the addition of linked read analyses promises to overcome the constraints of conventional next-generation sequencing in detecting clinically actionable SVs, thereby providing a more complete picture of the treatments available for patients with refractory malignancies.

## Additional files

Additional file 1: Supplemental Tables. **Table S1. ** Platform comparison. **Table S2.** Alignment metrics. **Table S3.** Summary metrics for linked read WGS. **Table S4.** Long Ranger SV results. **Table S5** LumPy SV summary. **Table S6.** CNV results. **Table S7.** SV phasing results for both metastatic sites. **Table S8.** De novo assembly statistics for both metastatic sites. (XLSX 50 kb)

Additional file 2: Supplemental Figures. **Figure S1. ** Overview of linked read sequencing. **Figure S2..** Tumor sample locations. **Figure S3.** Overview of SV processing pipeline. **Figure S4.** Method to phase structural variants. **Figure S5.** The landscape of somatic copy number alterations. **Figure S6.** Assessment of *FGFR2* copy number in primary and metastatic DGC by ddPCR. **Figure S7.** Barcode overlap plots of the 2.9-Mb genomic region surrounding the proto-oncogene *FGFR2*. **Figure S8.** Allele-specific barcode counts in *FGFR2* region of left metastasis. **Figure S9.** Complex breakpoint resolution for deletion using molecular barcode mapping. **Figure S10.** Complex breakpoint resolution for inversion using molecular barcode mapping. **Figure S11.** Complex breakpoint resolution for inversion in the left metastasis using molecular barcode mapping. **Figure S12.** De novo assembled contigs reconstruct rearrangement breakpoints in the right metastasis. **Figure S13.** In vitro gastric organoid model. **Figure S14.** Exogenous *FGFR2* expression in gastric organoids. (PDF 19353 kb)

Additional file 3: Frequently altered genes in TCGA gastric samples. (CSV 101 kb)

## Abbreviations

CNV: Copy number variant
ddPCR: Droplet digital PCR
DGC: Diffuse gastric cancer
FFPE: Formalin-fixed paraffin-embedded
*FGFR2*: Fibroblast growth factor receptor 2
HMW: High molecular weight
Indel: Insertion/deletion
SNV: Single nucleotide variant
SV: Structural variant
TCGA: The Cancer Genome Atlas
WGS: Whole genome sequencing
