# Supplementary material for: Linked read sequencing resolves complex genomic rearrangements in gastric cancer metastases
Source: Genome Med. 2017 Jun 19;9:57. doi: 10.1186/s13073-017-0447-8 (PMC5477353; doi:10.1186/s13073-017-0447-8)
Supplement: Supplementary file 2 — Figure S1. Overview of linked read sequencing. Figure S2.. Tumor sample locations. Figure S3. Overview of SV processing pipeline. Figure S4. Method to phase structural variants. Figure S5. The landscape of somatic copy number alterations. Figure S6. Assessment of FGFR2 copy number in primary and metastatic DGC by ddPCR. Figure S7. Barcode overlap plots of the 2.9-Mb genomic region surrounding the proto-oncogene FGFR2. Figure S8. Allele-specific barcode counts in FGFR2 region of left metastasis. Figure S9. Complex breakpoint resolution for deletion using molecular barcode mapping. Figure S10. Complex breakpoint resolution for inversion using molecular barcode mapping. Figure S11. Complex breakpoint resolution for inversion in the left metastasis using molecular barcode mapping. Figure S12. De novo assembled contigs reconstruct rearrangement breakpoints in the right metastasis. Figure S13. In vitro gastric organoid model. Figure S14. Exogenous FGFR2 expression in gastric organoids. (PDF 19353 kb) [file 13073_2017_447_MOESM2_ESM.pdf]

Figure S1

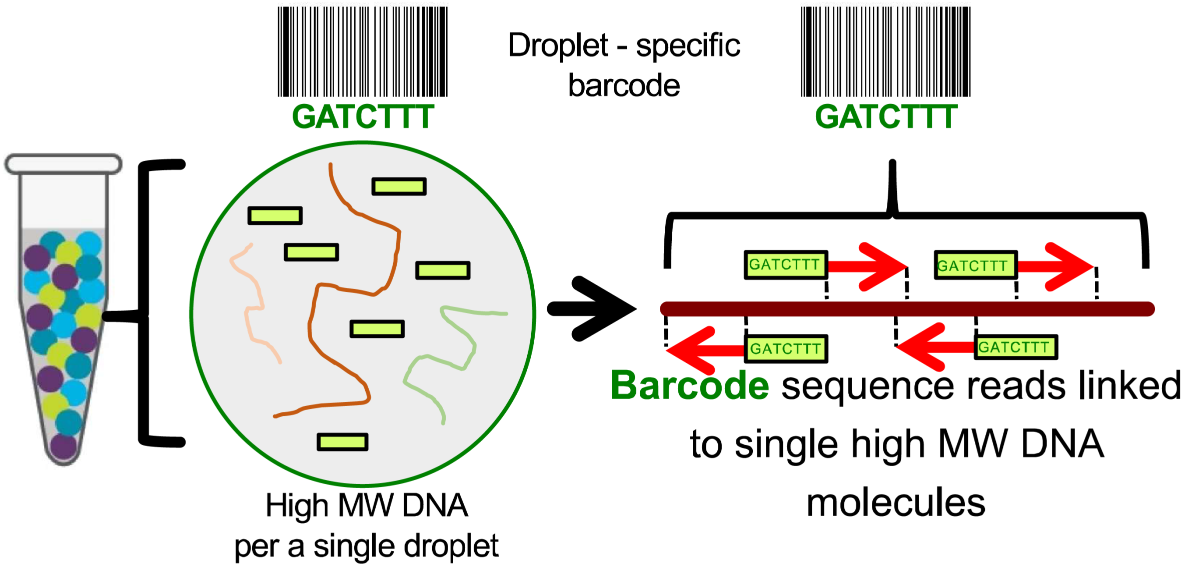

Figure S1. Overview of linked read sequencing. HMW DNA molecules are partitioned into droplets and labeled with unique barcodes; once labeled, the partitions are dissolved then sequencing libraries are prepared and sequencing is performed as usual. The barcode labels of individual sequence reads delineate the original HMW DNA molecules, and thus long range information is retained.

Figure S2

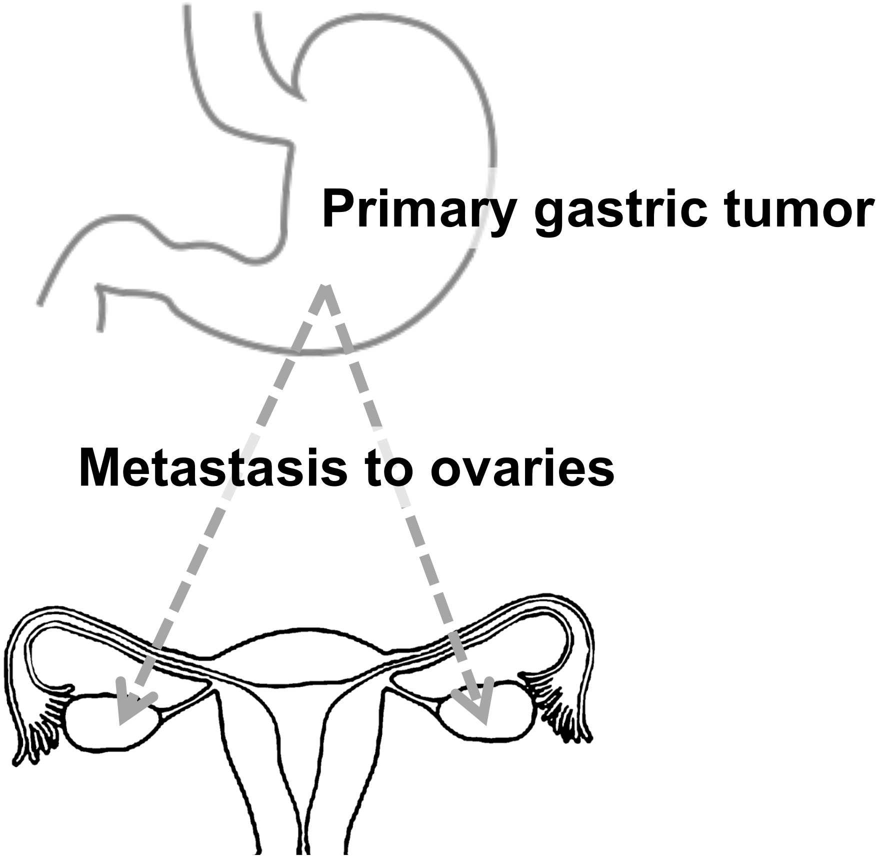

Figure S2. Tumor sample locations. The samples for this study were obtained from a patient with a primary diffuse gastric cancer tumor and metastases located in each ovary (Krukenberg tumors).

Figure S3

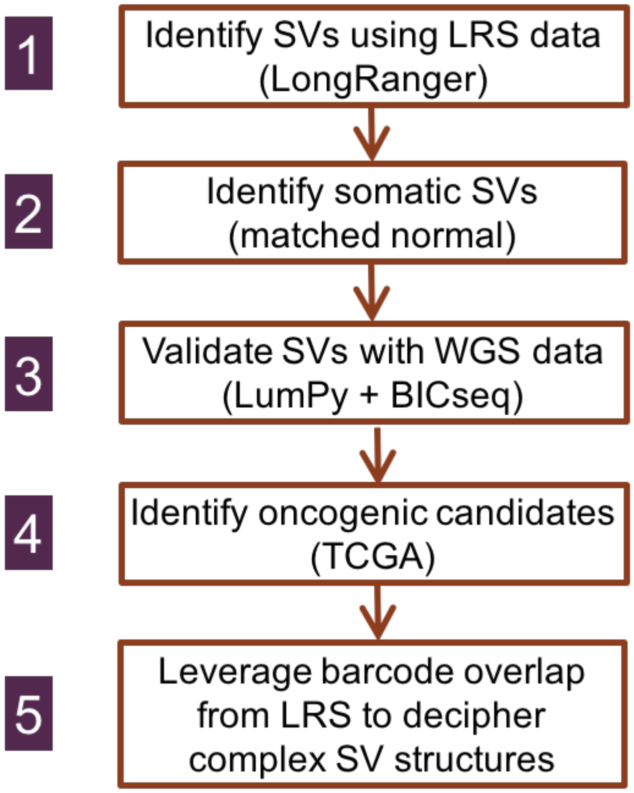

Figure S3. Overview of SV processing pipeline. SVs are identified using Long Ranger software which uses barcoded reads from linked read sequencing **(LRS)** to identify distant genomic regions with greater than expected levels of barcode overlap. Then, SVs are categorized as somatic if they do not occur in the normal sample, and undergo validation using SV callers designed for conventional TruSeq genome data. Putatively cancer-related SVs are determined based on their intersection with genes with a high occurrence in cancer, according to TCGA. Finally, clusters of SVs are identified, putatively representing complex structural rearrangements; barcoded read analyses can be leveraged to elucidate these complex events.

Figure S4

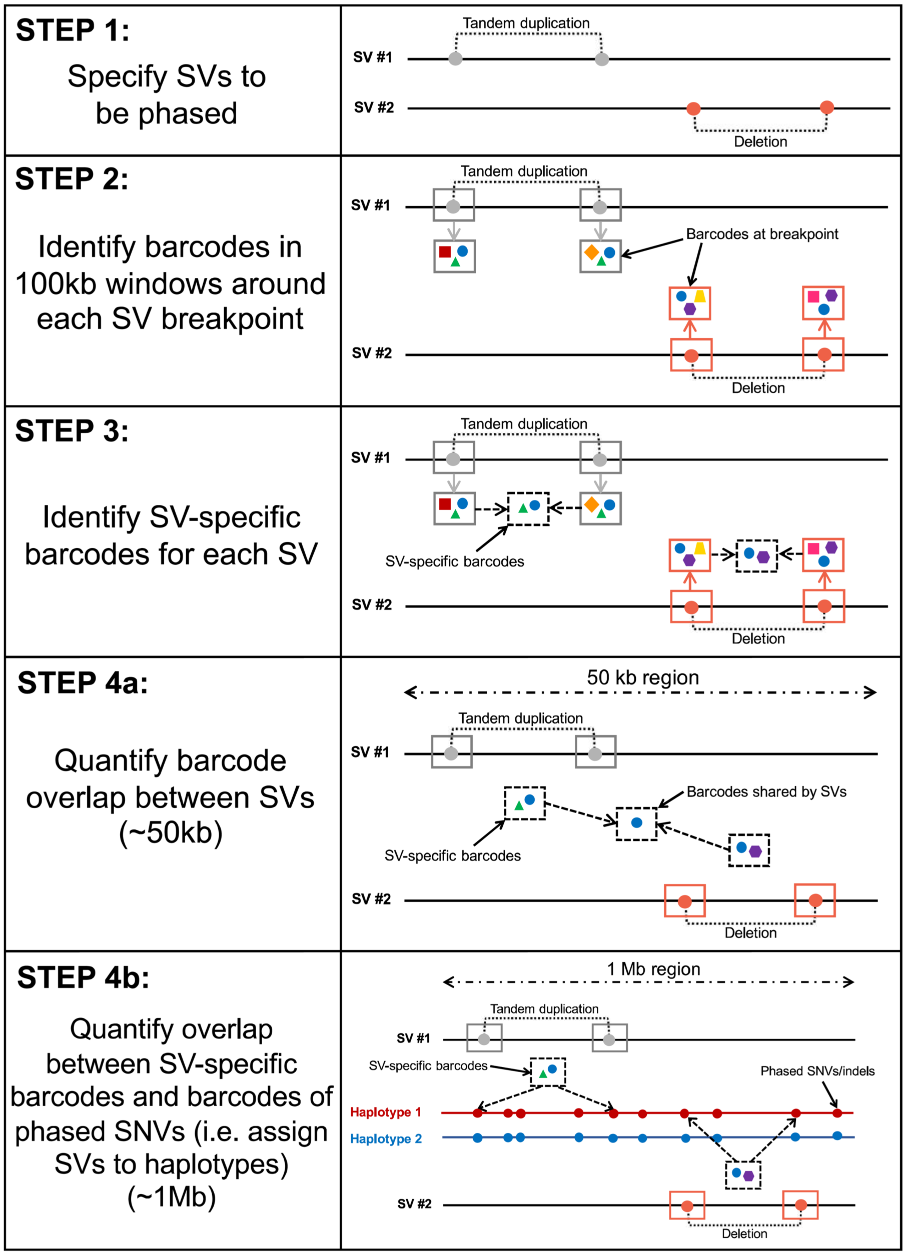

Figure S4. Method to phase structural variants. First, the genomic coordinates of the structural variants to be phased are specified by a structural variant caller (e.g. Long Ranger); each structural variant has two genomic breakpoints (Step 1). Barcodes are obtained for the reads that map in a 100 kb window around each SV breakpoint (Step 2). SV-specific barcodes are determined as those barcodes that occur at each SV breakpoint (Step 3); these SV-specific barcodes are then used to phase SVs. If SV-specific barcodes are shared between distinct SV events then these SVs are considered to have been present on the same HMW DNA molecule (average size: ~50 kb), and are therefore in *cis* (Step 4a). SVs can be phased across greater genomic distances by assigning each SV breakpoint to haplotyped phase blocks (average size: ~1 Mb) using barcodes assigned to phased heterozygous SNVs (Step 4b).

Figure S5

Outer track: Left ovary metastasis
Middle track: Right ovary metastasis
Inner track: Primary tumor

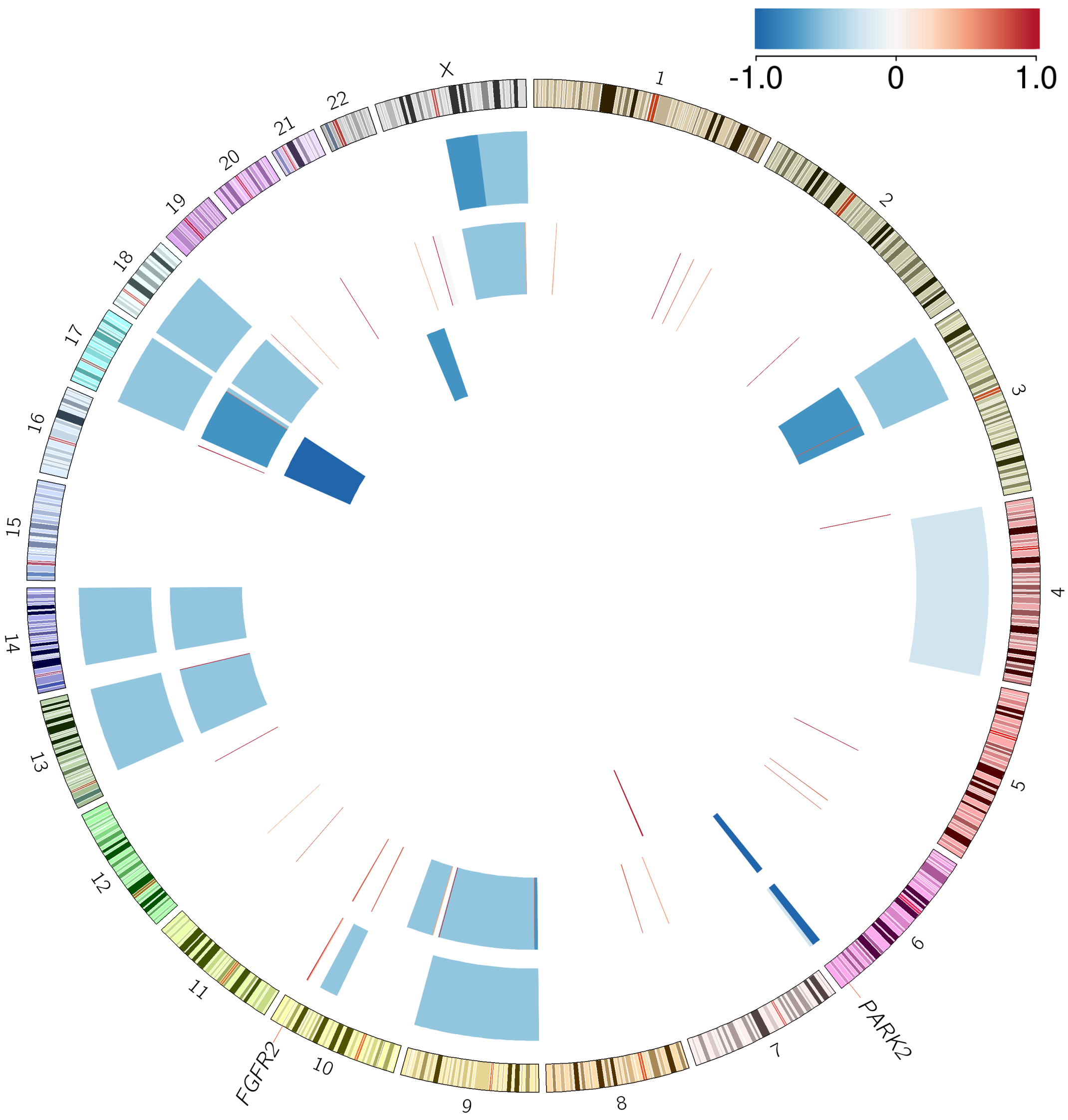

Figure S5. The landscape of somatic copy number alterations. Circos depiction of CNV calls from conventional WGS data using the BICseq CNV calling program. From outer to inner, the tracks represent CNV events in the left metastasis, right metastasis, and primary tumor. The data is presented as a tumor/normal ratio, scaled such that a value of zero signifies no change in the tumor compared to the normal. Red means gain or amplification, blue means loss or deletion.

Figure S6

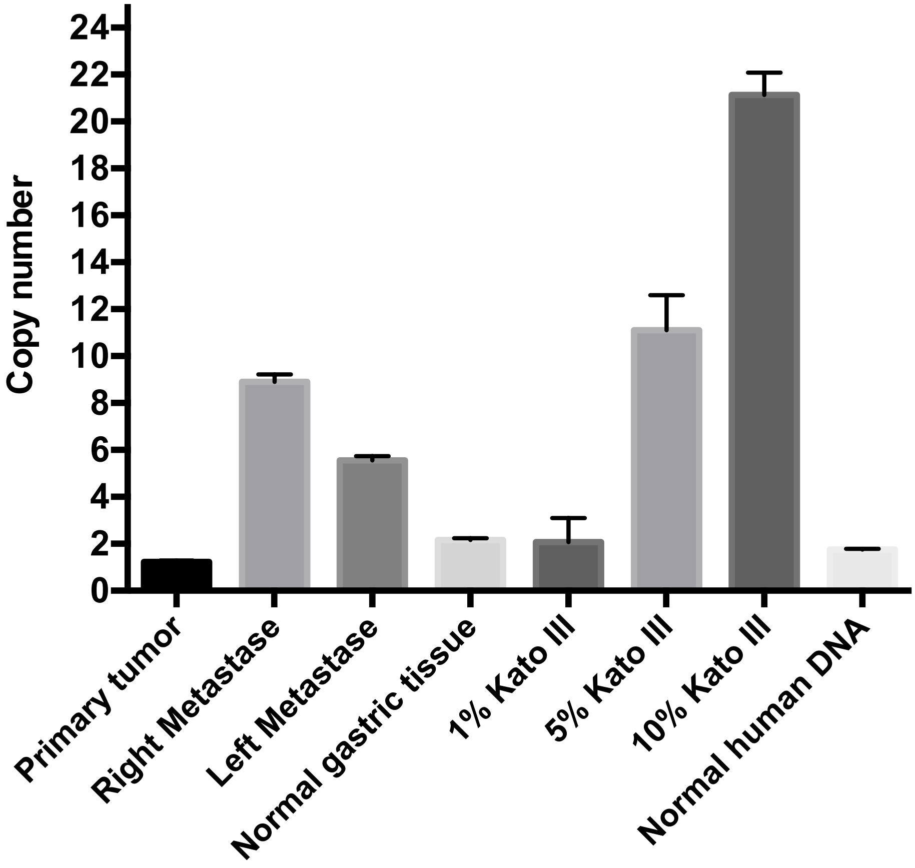

Figure S6. Assessment of *FGFR2* copy number in primary and metastatic DGC by ddPCR. *FGFR2* copy number per cell was estimated as the ratio of the *FGFR2* and reference *RPP30* copy number concentrations multiplied by two. Each sample was measured in triplicate. gDNA mixed with various amounts of Kato III and normal human gDNA (NA18507, Coriell) was used as a positive control.

Figure S7

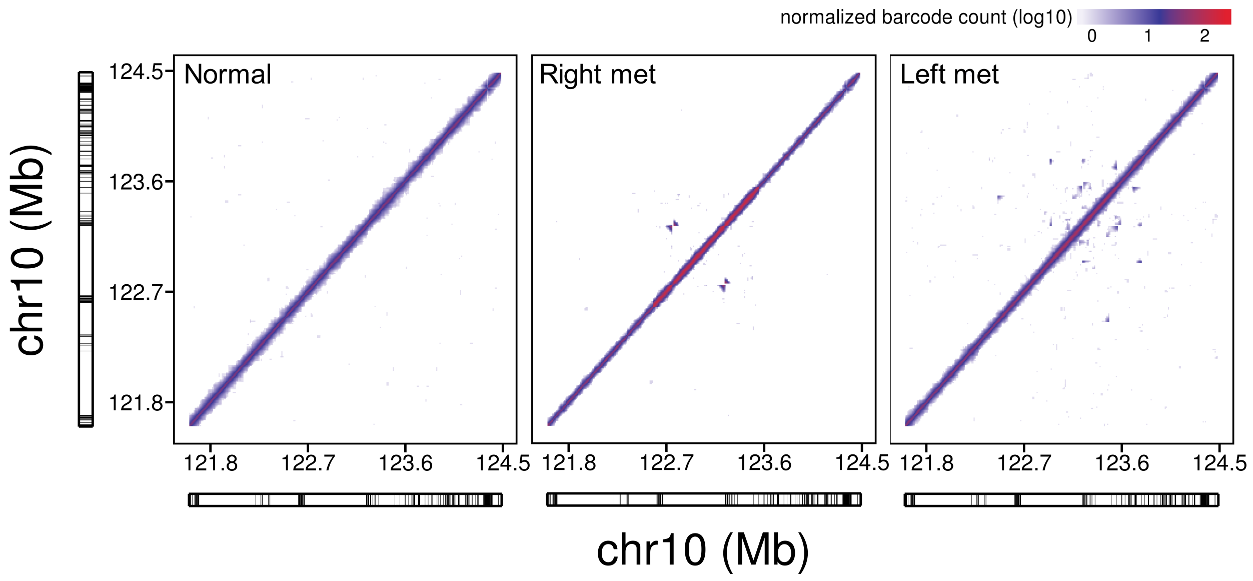

Figure S7. Barcode overlap plots of the 2.9 Mb genomic region surrounding the proto-oncogene *FGFR2*. The level of barcode sharing between 10 kb windows in a 2.9 Mb genomic region including *FGFR2* was determined for the normal sample, and the right and left metastatic samples. The highest level of overlap (red) is expected along the diagonal, while off-diagonal signals (red or blue) indicate the presence of structural variants.

Figure S8

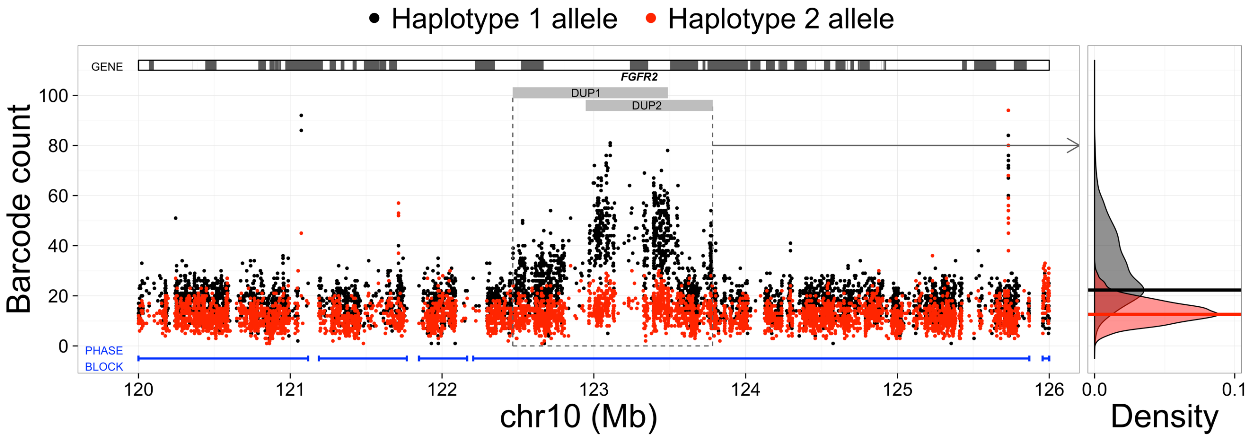

Figure S8. Allele-specific barcode counts in *FGFR2* region of left metastasis. Two distinct high-level amplifications occurred in this region, affecting one haplotype (haplotype 1; black).

Figure S9

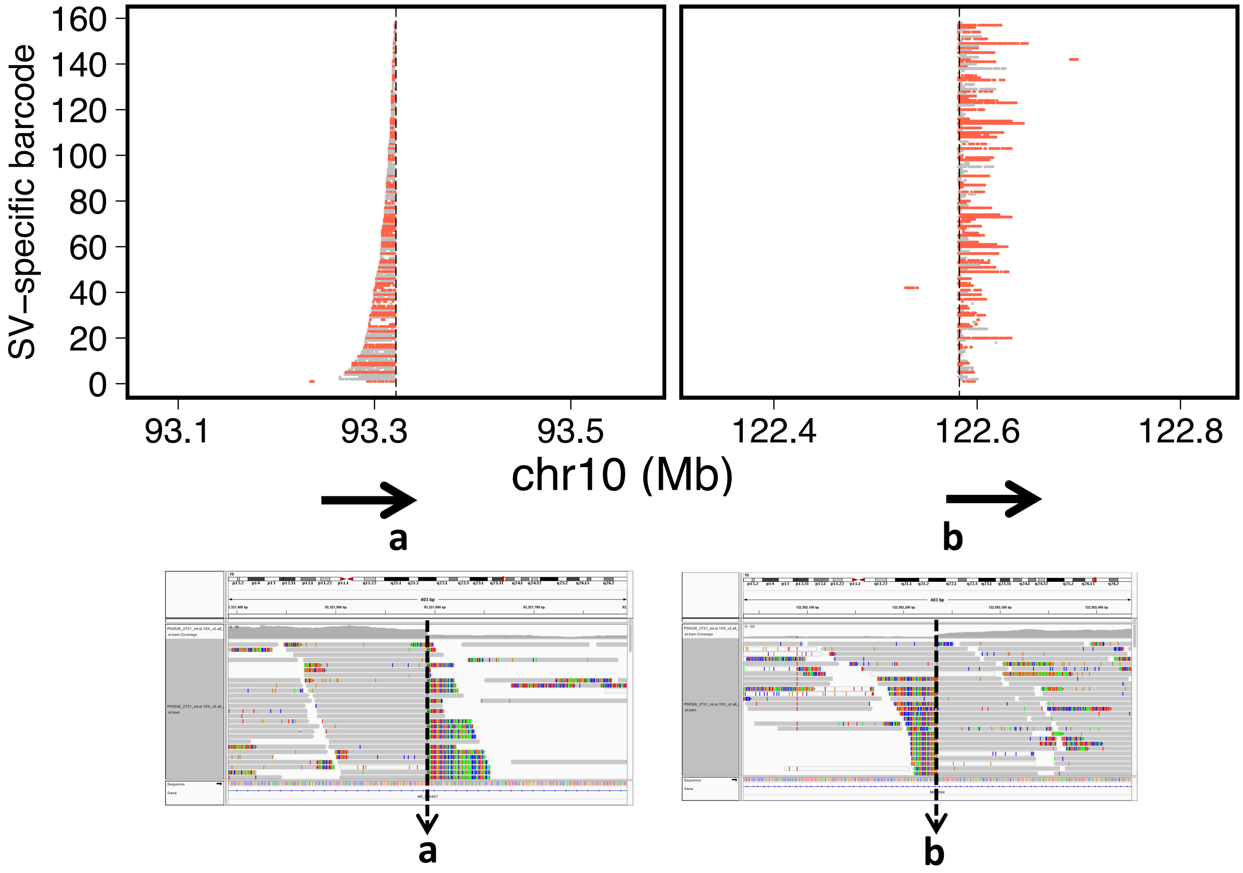

Figure S9. Complex breakpoint resolution for deletion using molecular barcode mapping. The SV-specific molecules for breakpoint 1 and breakpoint 2 of the deletion SV in the right metastasis are plotted according to the mapping location of molecular barcoded reads. Each row of the plot represents one SV-specific molecule, depicting how each SV-specific molecule spans the SV breakpoint. The arrow structure indicates breakpoint connection and directionality. IGV plots of the molecular breakpoints display soft-clip evidence.

Figure S10

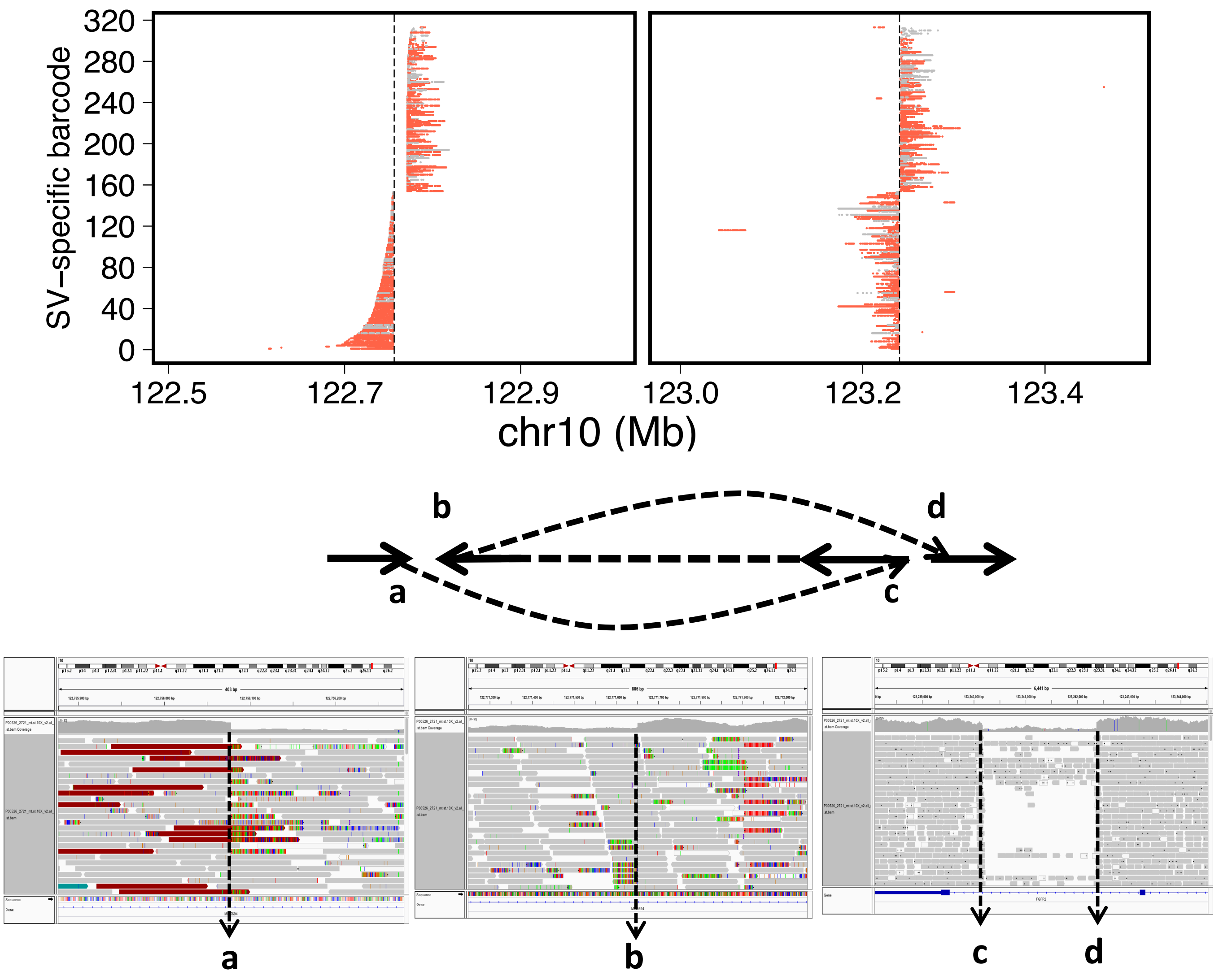

Figure S10. Complex breakpoint resolution for inversion using molecular barcode mapping. The SV-specific molecules for breakpoint 1 and breakpoint 2 of the inversion SV in the right metastasis are plotted according to the mapping location of molecular barcoded reads. Each row of the plot represents one SV-specific molecule, depicting how each SV-specific molecule spans the SV breakpoint. The arrow structure indicates breakpoint connection and directionality. IGV plots of the molecular breakpoints display soft-clip evidence.

Figure S11

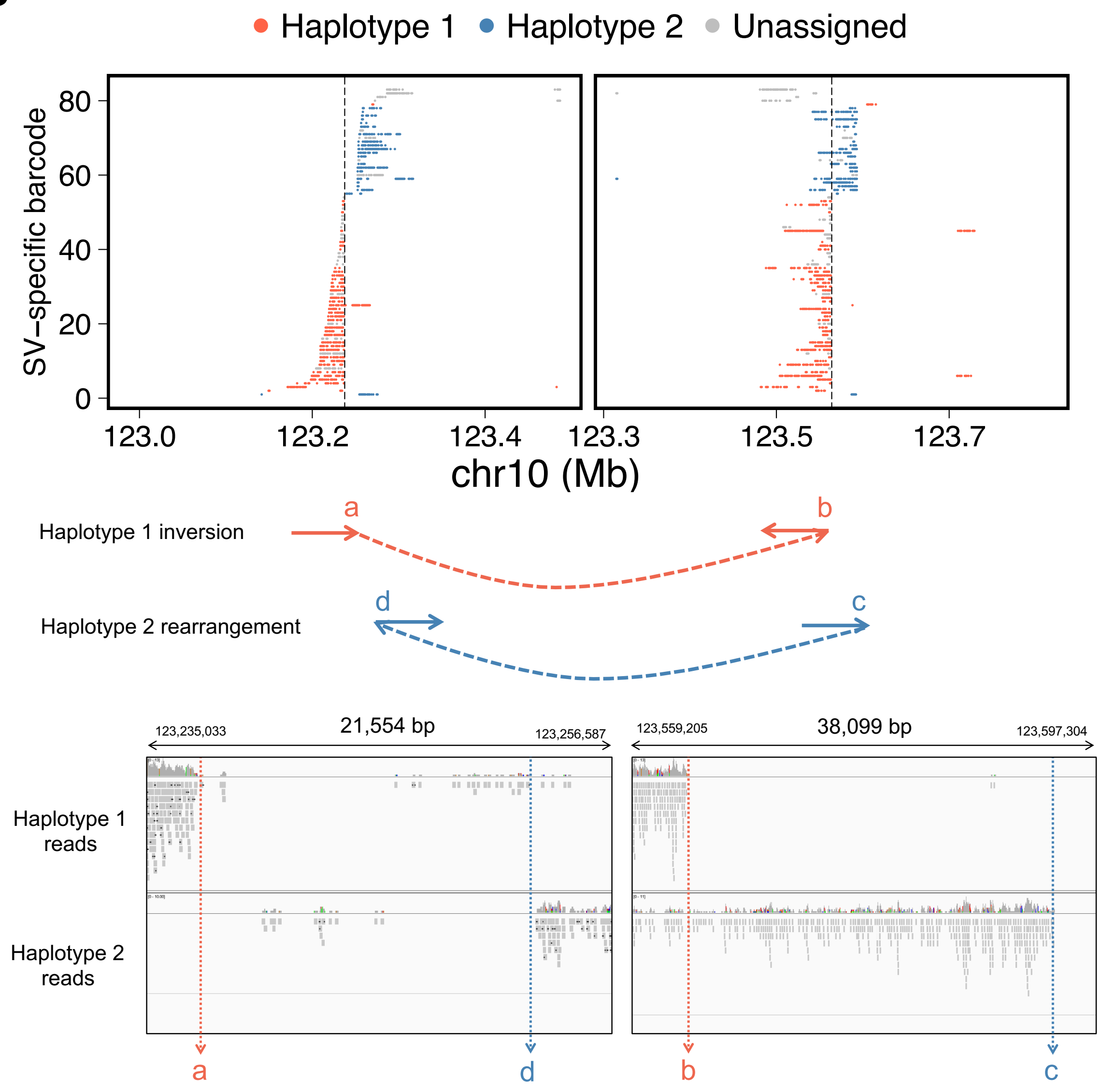

Figure S11. Complex breakpoint resolution for inversion in the left metastasis using molecular barcode mapping. The SV-specific molecules for breakpoint 1 and breakpoint 2 of the inversion SV in the left metastasis are plotted according to the mapping location of molecular barcoded reads. Each row of the plot represents one SV-specific molecule, depicting how each SV-specific molecule spans the SV breakpoint. The arrow structure indicates breakpoint connection and directionality. IGV plots of the molecular breakpoints display soft-clip evidence.

Figure S12

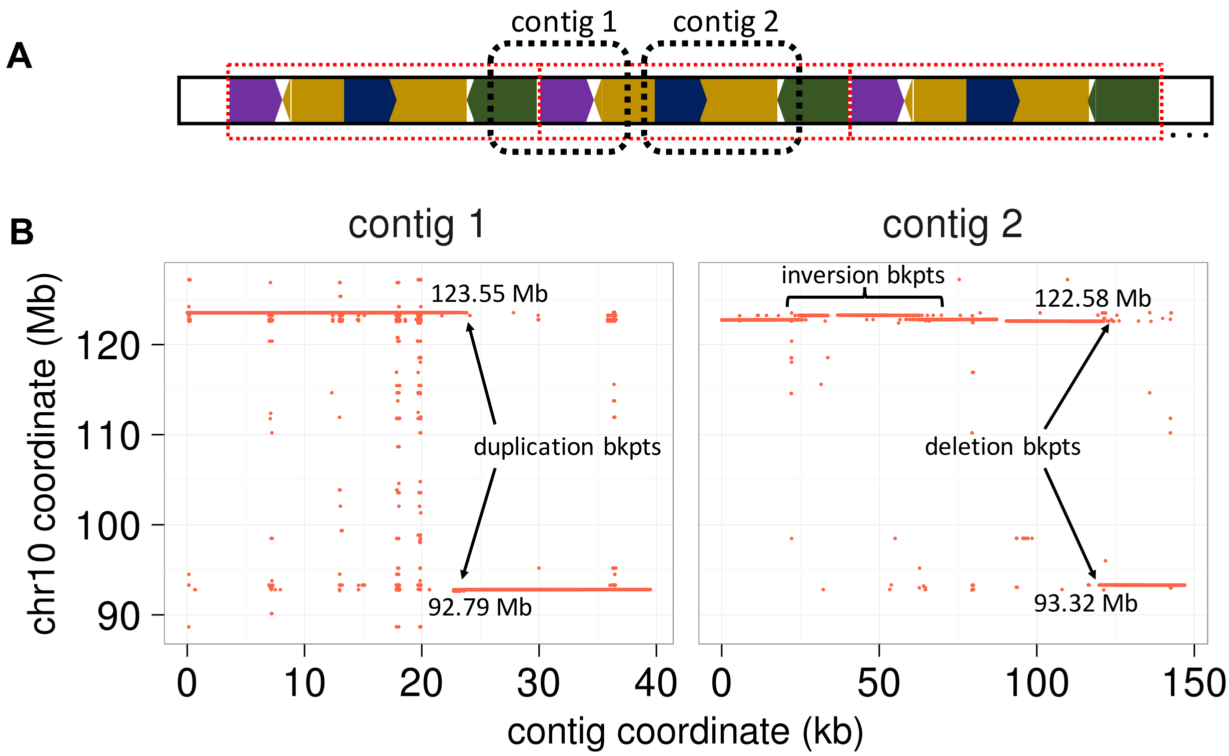

Figure S12. ***De novo*** **assembled contigs reconstruct rearrangement breakpoints in the right metastasis.** Contig 1 and contig 2 were assembled by supernova from reads with SV-specific barcodes (i.e. SV-specific reads). (A) Contig 1 contained the duplication breakpoint and contig 2 contained the deletion and inversion breakpoints. (B)Plotted here is the mapping location of each SV-specific read on the contig and on the genome, resulting in a depiction of the contig structure.

Figure S13

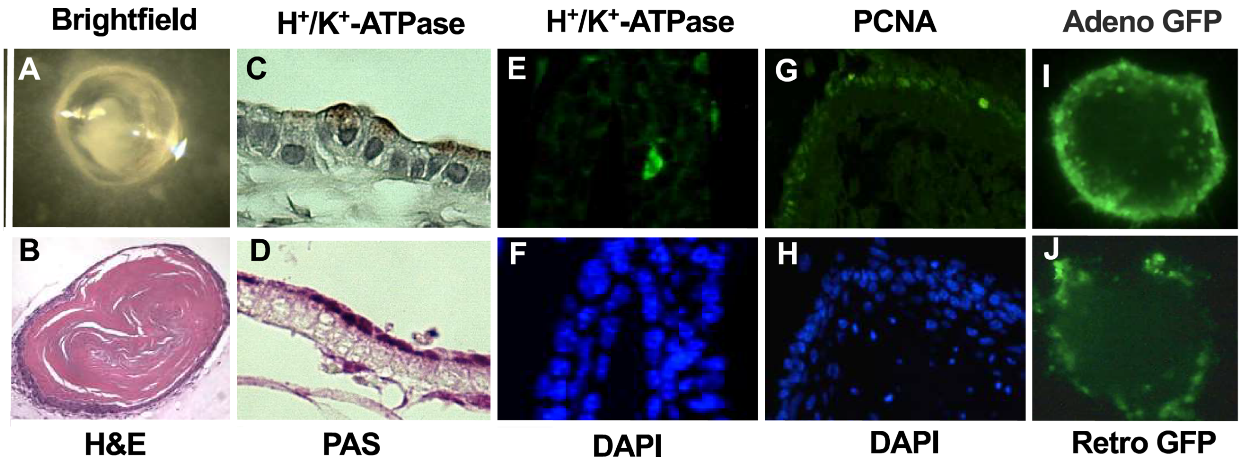

Figure S13. ***In vitro* gastric organoid model.** (A) Gastric organoids grown in a collagen matrix visualized under light microscopy. (B) H&E staining of gastric organoids reveals a single-cell polarized epithelial layer. (C&E) Immunostaining with antibody against H^+/K^+-ATPase confirms the presence of H^+-producing parietal cells. (D) Mucin-producing cells are confirmed by Periodic Acid Schiff-positive cells (PAS). (F&H) DAPI staining indicates viable cell nuclei in wildtype gastric organoids. (G) Proliferative cells are identified by positive immunofluorescence for PCNA in wildtype gastric organoids. Gastric organoids are infected by adenovirus GFP (I) and by retrovirus GFP (J).

Figure S14

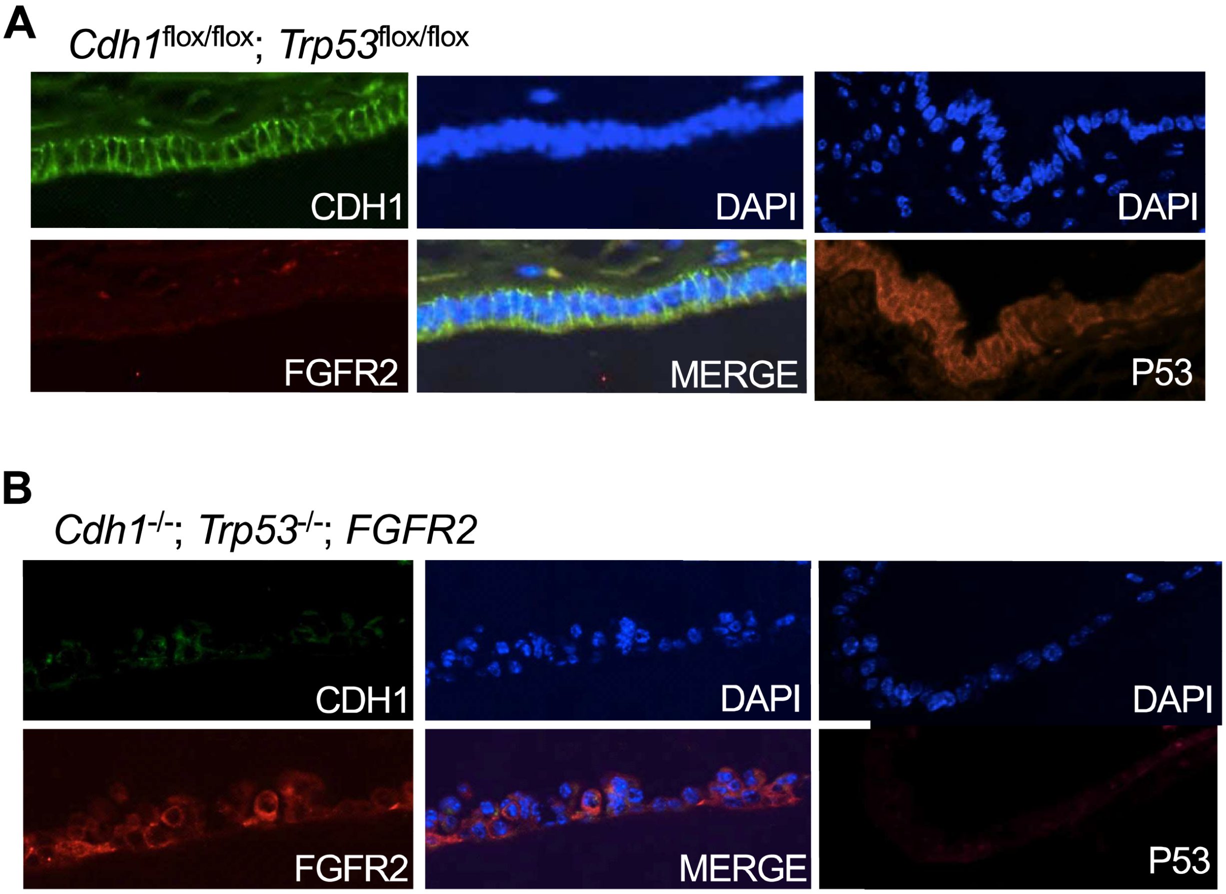

Figure S14. Exogenous *FGFR2* expression in gastric organoids. (A) *Cdh1$^{flox/flox}$; Trp53$^{flox/flox}$* gastric organoids imaged by immunofluorescence with antibodies against Cdh1, FGFR2 or Trp53. The organoids were infected with Fc-expressing adenovirus as a control. (B) *Cdh1$^{-/-}$; Trp53$^{-/-}$, FGFR2* gastric organoids were imaged by immunofluorescence with antibodies against Cdh1, FGFR2, or Trp53. The organoids were generated by infecting *Cdh1$^{flox/flox}$; Trp53$^{flox/flox}$* with cre-recombinase adenovirus and retroviral *FGFR2.*
